# Determining Ligand Binding and Specificity Within the β_2_-Integrin Family with a Novel Assay Platform

**DOI:** 10.3390/biom15020238

**Published:** 2025-02-07

**Authors:** Carla Johanna Sommer-Plüss, Céline Leiggener, Elira Nikci, Riccardo Vincenzo Mancuso, Said Rabbani, Christina Lamers, Daniel Ricklin

**Affiliations:** 1 Molecular Pharmacy Research Group, Department of Pharmaceutical Sciences, University of Basel, Klingelbergstrasse 50, 4056 Basel, Switzerland; 2 Institute for Drug Development, Faculty of Medicine, University of Leipzig, Brüderstraße 34, 04103 Leipzig, Germany

**Keywords:** β_2_-integrin receptors, complement, CR3, CR4, LFA-1, CD11d/CD18, drug discovery

## Abstract

The family of the β_2_-integrin receptors is critically involved in host defense and homeostasis, by mediating immune cell adhesion, migration, and phagocytosis. Due to their key roles in immune surveillance and inflammation, their modulation has been recognized as an attractive drug target. However, the development of therapeutics has been limited, partly due to the high promiscuity of endogenous ligands, their functional responses, and gaps in our understanding of their disease-related molecular mechanisms. The delineation of the molecular role of β_2_ integrins and their ligands has been hampered by a shortage of validated assay systems. To facilitate molecular and functional studies on the β_2_-integrin family, and to enable screening of modulators, this study provides a uniform and validated assay platform. For this purpose, the major ligand-binding domains (αI) of all four β_2_ integrins were recombinantly expressed in both low- and high-affinity states. By optimizing the expression parameters and selecting appropriate purification tags, all αI-domain variants could be produced with high yield and purity. Direct binding studies using surface plasmon resonance (SPR) confirmed the expected activity and selectivity profiles of the recombinant αI domains towards their reported ligands, validating our approach. In addition, the SPR studies provided additional insights into ligand binding, especially for the scarcely described family member CD11d. Alongside characterizing endogenous ligands, the platform can be employed to test pharmacologically active compounds, such as the reported β_2_-integrin antagonist simvastatin. In addition, we established a bead-based adhesion assay using the recombinant αI domains, and a cell-based adhesion assay underlining most findings generated with the isolated αI domains. Interestingly, the binding of ligands to the recombinant α_D_I is not dependent on divalent cation, in contrast to the full integrin CD11d/CD18, suggesting a binding mode distinct of the metal ion-dependent adhesion site (MIDAS). The setup highlights the applicability of recombinant αI domains for first screenings and direct or competitive interaction studies, while the full integrin is needed to validate those findings.

## 1. Introduction

Host defense and immune surveillance critically relies on a finely tuned interplay between cellular and humoral partners. Especially, the leucocyte-specific β_2_-integrin family plays a particularly important role in leukocyte functions, mediating rolling, adhesion, extravasation, and phagocytosis [1,2]. Integrins are heterodimeric surface receptors composed of an α and β subunit, which are non-covalently connected at the headpiece via interactions of the β propeller of the α subunit and the I-like domain from the β subunit [3,4]. The β_2_-integrin family shares a common β subunit (β_2_ or CD18), while the α subunit defines the specific member and ligand specificity. The β_2_-integrin family consists of four members. Lymphocyte function-associated antigen 1 (LFA-1; also termed α_L_β_2_ or CD11a/CD18) is a component of the immune synapse and mediates leukocyte migration and adhesion, mainly by interacting with intercellular adhesion molecules (ICAM-1, -2, -3, -4, and -5) [5,6,7,8]. Complement receptor 3 (CR3; α_M_β_2_, CD11b/CD18, Mac-1) and complement receptor 4 (CR4, α_X_β_2_, CD11c/CD18, p150,95) enable the phagocytic removal of pathogens and other cells/particles upon opsonization by the complement system. As a major host defense mechanism, the complement system recognizes foreign or altered cell surfaces and activates an enzymatic cascade that leads to the activation of the plasma protein C3 with subsequent covalent deposition of the C3b fragment [9,10]. This opsonin can directly mediate effector functions by building the platform for C3 convertase and C5 convertase assembly but is also processed to the iC3b and C3dg fragments that interact with receptors, e.g., the β_2_ integrins CR3 and CR4 [11,12,13,14,15,16,17]. CD11d/CD18 (α_D_β_2_) is the most recently discovered member and information on its ligand-binding and functional profile is scarce, but reported to be mostly ICAM-3, VCAM-1 and extracellular matrix components [18,19,20].

Overall, integrins share a common general structure [1], and for ligand binding, the headpiece, and in the case of β_2_ integrins, the I-domain, an inserted von Willebrand factor A (vWFA) domain of approximately 200 amino acids, has been identified as a ligand recognition site [21,22]. The four members of the β_2_-integrin family show considerable sequence identity within their αI domain, with especially high similarity between α_D_I, α_M_I, and α_X_I (55–60% sequence identity), while the α_L_I domain is more distinct and shares only one-third of the sequence with the other family members [18].

Owing to their central role in orchestrating tissue homeostasis and immune responses, β_2_ integrins were found to be involved in various autoimmune, inflammatory, and age-related diseases [23,24,25]. Genetic defects in the ITGB2 gene, coding for CD18, results in a disorder marked by recurrent bacterial infections known as leukocyte adhesion deficiency (LAD) type I [26,27]. Furthermore, LFA-1 has been identified as a major contributor to dry eye syndrome, with the LFA-1 antagonist lifitegrast being approved for treatment. In addition, LFA-1 and CR3 have been associated with other autoimmune disorders, such as systemic lupus erythematosus [28,29] and psoriasis, where an anti-LFA-1 antibody (Efalizumab) had been marketed but was withdrawn due to safety concerns. Interestingly, CR3 is involved in synaptic pruning and regulation of Aβ-induced neuroinflammation [30,31], and has been discussed as a prominent contributor to the development of neurological disorders, including schizophrenia and Alzheimer’s disease [32]. Similarly, an association of LFA-1 with Alzheimer’s disease has been reported [33]. More recently, a CR3 agonist has been investigated for the treatment of cancer [34,35]. Despite their documented disease involvement and recognition as promising therapeutic targets, drug development for β_2_ integrins has remained limited [21,22]. The reasons for this are safety concerns due to their role in host defense, an often-incomplete understanding of the specific pathological role of individual family members, and the complexity of integrin receptors as drug targets [22,26].

Integrins generally show a conformational complexity with three distinct conformations (i.e., extended-open, extended closed, bent-closed), which have been linked to so-called “outside-in” and “inside-out” signal transduction. Hereby “open” and “closed” are generic terms for high- and low-affinity states of the I domain for ligand binding. Those conformations are regulated within the I domains by the metal ion-dependent adhesion site (MIDAS) and the socket for isoleucine (SILEN) [36], impacting the position of the α7 helix. On living cells, integrins are normally present in a bent-closed state with a low affinity for ligands [26,37], as the SILEN motif stabilizes the closed I-domain conformation by interaction with an isoleucine within the α7 helix [38]. Until recently, it was postulated that upon intra- and/or extracellular triggers, integrins open to an extended, high-affinity conformation for ligands, in a switchblade-like motion through binding of the cytoskeletal protein talin or kindlin to the cytoplasmic tail [3,39]. This process, called inside-out signaling, enables subsequent ligand binding. It was postulated that during the talin- and kindlin-binding-induced conformational change of the integrin, the αI domain would also undergo structural changes, inducing an open state with high affinity for ligands [3,40]. Indeed, the binding affinity of the high affinity state of the I domain was reported to be 2000-fold increased [41]. This results in an activated integrin transmitting a signal inside the cell using conformational changes induced upon ligand binding, the so-called outside-in signaling. Interestingly, this high-affinity form of the I domain can be generated in recombinant, isolated αI domains using stabilizing mutations [14,18,38].

Opposing this hypothesized mechanism, Li et al. showed in a recent study, using a FRET-based setup, that binding of an extracellular ligand to the integrins α_5_β_1_ and α_4_β_1_ is initiating integrin activation, prior to interaction with cytoskeletal proteins talin and kindlin. Furthermore, they concluded from their results that extension and headpiece opening are a concerted mechanism and this mechanism might be general for all integrins [41].

An additional layer of complexity for the development of targeted therapeutics is the broad ligand spectrum. In case of CR3, it is exceptionally large with nearly 100 ligands reported, ranging from ECM-components over ICAMS to complement opsonins [21]. The delineation of the ligand’s functional responses and selectivity has been slow and hampered, partly due to a shortage of sensitive and comparable assay formats. Especially, molecular interaction data are scarce for both endogenous ligands and synthetic modulators or not comparable due to the use of different setups (i.e., full integrin on cells vs. isolated ectodomains or I domains), recombinant constructs, and used expression systems. For the β_2_-integrin family, recombinant αI domains are often used in assays, but rarely in direct comparison of all four family members produced under the same conditions. Consequently, there is an unmet need for an improved assay platform that enables a quantitative and comparative insight into the ligand-binding profiles of β_2_-integrins and facilitates the screening and optimization of target-specific modulators for research and therapy.

In this study, we report an assay platform consisting of recombinant αI domains of all four β_2_ integrins, as wild type (WT), imitating the “closed” formation, and high-affinity (HA) “open” variants. We validated these recombinant αI domains successfully in surface plasmon resonance (SPR) interaction studies with the literature-reported ligands ICAM-1 and complement opsonins C3b, iC3b, and C3dg. For investigating the role of the β_2_-integrin αI domains in adhesion, we established a beads-based assay utilizing the recombinant I domains, and set up an adhesion assay using HEK293 cells stably transfected with CR3, CR4, or α_D_β_2_. These assays allow us to study β_2_-integrin ligand binding and, thereby, we gain valuable insight into the complexity of the β_2_-integrin family and investigate their potential as therapeutic targets.

## 2. Materials and Methods

### 2.1. Plasmids, Cells, Antibodies, and Purified Proteins

Purified complement proteins (C3, C3b, iC3b, C3d, FB, FD, FH, and FI) were purchased from Complement Technology, Inc. (Tyler, TX, USA). The ICAMs were commercially available from Sino Biological as recombinant protein containing His- and/or human Fc-tag (ICAM-1, cat: 10346-H03H; ICAM-2, cat: 10332-H03H, ICAM-3, cat: 10333-H03H; ICAM-4: cat: 13327-H02H; ICAM-5, cat: 16050-H08H). A plasmid containing the α_M_I sequence [42] and recombinant CR1 (CCP15-17) [43] were kindly provided by Christoph Q. Schmidt (Ulm University, Germany). Plasmids containing the α_L_I, α_X_I and GST sequences were obtained from Addgene (Plasmids #8630 and #8633 both contributed by Timothy Springer, Harvard University, USA, plasmid #42049 contributed by Kumiko Ui-Tei, University of Tokyo, Japan). A plasmid containing the α_D_I sequence was purchased from ATG:Biosynthetics GmbH (Merzhausen, Germany) and primers were ordered at Microsynth AG (Balgach, Switzerland). HEK293T cells containing CR3 and CR4 were kindly provided by Carla de Haas and Suzan Rooijakkers (UMC Utrecht, The Netherlands), and HEK293 cell line containing CD11d/CD18 was kindly provided by Valentin Yakubenko (East Tennessee State University, Johnson City, TN, USA). Antibodies were purchased from Thermo Fisher Scientific (Waltham, MA, USA): anti-CD11a clone R7.1 without label and FITC labeled, anti-CD11b clone M1/70 without label and APC labeled, anti-CD11c clone 3.9 without label and APC labeled, and anti-CD18 clone 6.7, FITC labeled. Due to the shortage of a commercially available monoclonal antibody against CD11d, a polyclonal antibody (PA5103445, Invitrogen, Waltham, MA, USA) was used.

### 2.2. Protein Expression and Purification

The CR3 α_M_I, CR4 α_X_I, LFA-1 α_L_I, and CD11d α_D_I domains were recombinantly expressed as WT and HA form in *E. coli* and purified by affinity chromatography. To obtain the HA variant, the following mutations have been used: CR3 α_M_I p.I332G, CR4 α_X_I p.I333G, LFA-1 α_L_I p.K312C/K319C, and CD11d α_D_I p.I332G. The corresponding mutations were based on the published literature [14,38,44], or in the case of CD11d α_D_I, on sequence homology analysis with CR3 α_M_I and CR4 α_X_I (Supplementary Figure S1). The sequences encoding for the individual αI domain and a His-tag were fused either to a GST-Tag or directly cloned into the expression vector pET15b. After sequence confirmation, plasmids were transformed into the *E. coli* expression strain BL21(DE3) (Agilent, Santa Clara, CA, USA) using the heat shock method. Colonies growing on culture plates containing 100 µg/mL ampicillin were screened for expression levels prior to a scale-up of the best-expressing colony. The *E. coli* were cultured in terrific broth containing 100 µg/mL ampicillin for 8 h at 37 °C, and cooled down to 18 °C before induction of the protein expression with 0.5 mM IPTG for 65 h. Cells were harvested by centrifugation (5400× *g*, 4 °C, 25 min) before resuspension in binding buffer (50 mM NaH_2_PO_4_, 300 mM NaCl, 40 mM imidazole, pH 7.5) and cell lysis using a Pressure Cell homogenizer (FPG12800; Homogenising Systems, Stansted, UK). The cellular debris were pelleted by centrifugation for 30 min at 15,500× *g* and 4 °C followed by ultracentrifugation for 30 min at 57,900× *g* and 4 °C.

Proteins were purified using an ÄKTA pure system (Cytiva, Marlborough, MA, USA) on a HisTrap FF 5 mL column (Cytiva, Marlborough, MA, USA). After loading of the filtered lysate, the column was washed 10 times with binding buffer and proteins were eluted in elution buffer (50 mM NaH_2_PO_4_, 300 mM NaCl, 250 mM imidazole, pH 7.5). Protein-containing fractions were pooled and imidazole was removed by dialysis against PBST (8.0 g/L NaCl, 0.2 g/L KCl, 0.2 g/L KH_2_PO_4_, 1.15 g/L anhydrous Na_2_HPO_4_, 0.1% Tween-20, pH 7.4).

To examine whether the produced proteins were correctly folded, nano differential scanning fluorimetry (nanoDSF) experiments were performed using a Prometheus instrument (Nanotemper Technologies GmbH, Munich). Protein samples were diluted in PBST to 0.5 mg/mL and 1mM MgCl_2_ was added to each sample. The samples were measured in triplicates in high-sensitivity capillaries. While heating the samples from 20–70 °C at a rate of 1 °C/s, changes of intrinsic fluorescence intensity by tryptophan and tyrosine at 330 nm and 350 nm, respectively, and changes in fluorescence intensity ratio (350:330 nm) were measured (Supplementary Figure S2).

### 2.3. Interaction Analyses

The interaction between the αI domains with their natural ligands was analyzed by SPR on a Biacore T200 instrument (Cytiva) at 25 °C. HBST buffer (10 mM HEPES, 150 mM NaCl, 0.005% Tween-20, pH 7.4) supplemented with either 1 mM MgCl_2_, 1 mM MgCl_2_ and 1 mM MnCl_2_, or 5 mM EDTA was used as running and sample buffer. For the interaction with C3 fragments, a CM5 sensor chip (Cytiva) was prepared based on previous reports [45]. C3b was immobilized by amine reactive immobilization. The carboxymethyl dextran surface was activated using 100 mM N-hydroxysulfosuccinimide (s-NHS), 50 mM 2-(N-morpholino)ethanesulfonic acid (MES), and 400 mM 1-ethyl-3-(3-dimethylaminopropyl)carbodiimide (EDC) for 7 min. C3b (5 µg/mL in 10 mM sodium acetate pH 5.0) was injected until a surface density of about 100 RU was reached. After deactivating the surfaces with 1 M ethanolamine for 7 min, a solution containing 0.5 µM factor B (FB) and 0.1 µM factor D (FD) was injected for 3 min at 5 µL/min. Immediately after, 1 µM C3 was injected for physiological deposition of C3b on the sensor chip. The FB/FD and C3 injections were repeated until C3b reached a surface density of about 5000 RU. The conversion of C3b to iC3b was achieved by injecting a solution containing 1 µM factor H (FH) and 0.1 µM factor I (FI) for 5 min at 5 µL/min. C3dg was generated by co-injecting 1 µM CR1 and 0.1 µM FI for 6 min at 5 µL/min. ICAM-1, ICAM-2, and ICAM-3 were immobilized on a CM5 sensor chip via amine reactive coupling. The chip was activated as described above and ICAMs were injected at a concentration of 10 µg/mL in 10 mM sodium acetate pH 5.0 until the desired surface density was reached (ICAM-1 & ICAM-2 around 3200 RU, VCAM-1 2100 RU) after which the flow cells were deactivated with 1 M ethanolamine.

For all interaction analyses, a non-immobilized flow cell was used as reference surface and in each dilution series, buffer blanks were included to allow double referencing. Displayed in the figures are representative SPR sensorgrams of multiple repetitions (n = 3–6). For kinetic analysis, a dilution series of the αI domains was injected on the surfaces for 120 s at a flow rate of 10 µL/min. After a dissociation time of 120 s, 5 mM EDTA was injected for 60 s to regenerate the surface. For competitive assays, a fixed concentration of the αI domains (5 µM) was preincubated with a dilution series (100 µM–3.125 µM) of simvastatin before injection. Data were processed using the Biacore T200 Evaluation Software (version 3.1, Cytiva, Marlborough, MA, USA). Association and dissociation rate constants (*k_a_*, *k_d_*) were fitted using a Langmuir 1:1 model, and the equilibration constant *K_D_* was calculated.

### 2.4. Bead-Based Adhesion Assay

A V-well-adhesion assay was adapted from a published protocol [46]. V-well plates were coated with ligands (iC3b, ICAM-1, or IgG as control) at a concentration of 10 µg/mL in coating buffer (20 mM Tris, 150 mM NaCl, pH 8.0) overnight at 4 °C. The plates were incubated with 200 µL blocking buffer (50 mM Tris, 150 mM NaCl, 1.5% BSA, pH 7.4) at 37 °C for 90 min to block non-specific binding. Per V-well, 5 µL protein G-coated fluorescent PAK Blue particles (Spherotech, Lake Forest, IL, USA) were coated for 2 h at room temperature on a rotating wheel with 20 µg/mL anti-His antibody (clone HIS.H8, Life Technology, Waltham, MA, USA). After washing with PBST, the beads were incubated overnight at 4 °C with 20 µg/mL of His-tag containing αI domains. The beads were washed with binding buffer (50 mM Tris, 150 mM NaCl, 1.5% BSA, 2 mM MgCl_2_, 2 mM MnCl_2_, 5 mM D-glucose, pH 7.4) and analyzed for adhesion at this state or incubated with 5 mM EDTA (MIDAS control) or anti-CD11a/CD11b/CD11c/CD11d antibodies (inhibition control) for 40 min at 37 °C. To analyze adhesion, the beads were transferred to the V-wells, incubated for 10 min at room temperature, and centrifuged at 200× *g* for 10 min at room temperature with brake off. Non-adherent beads that accumulated at the bottom of the V-well were quantified using an Infinite M200 Pro plate reader (Tecan, Männedorf, Switzerland) with excitation at 566 nm and emission at 671 nm. Statistical analysis has been calculated with GraphPad Prism 10.2.0, with an unpaired *t*-test from mean and standard deviation.

### 2.5. Transfected HEK Cell Adhesion Assay

Wild-type HEK293 cells and stably transfected HEK293 cell lines expressing either CR3 (CD11b/CD18), CR4 (CD11c/CD18), or CD11d/CD18 were cultured in high-glucose Dulbecco’s Modified Eagle’s Medium (DMEM, D5796 Sigma-Aldrich, St. Louis, MO, USA) supplemented with 10% heat-inactivated fetal bovine serum (FBS) and 1% penicillin/streptomycin. Cells were cultured in a humidified incubator at 37 °C and 5% CO_2_.

For CR3- and CR4-expressing HEK293 cells, CD11b/CD11c and CD18 cDNA were cloned in the dual promotor lentiviral vectors RP137 (BIC-PGK-Zeo) and RP139 (BIC-PGK-Puro), respectively. First, CD18 was stably expressed in HEK293 cells; subsequently, these cells were used for stable expression of CD11c. HEK293 cells were stably transfected with CD11d/CD18 as published earlier [18]. Expression of receptors was verified by selection antibiotics (zeocin, hygromycin, and puromycin), flow cytometry, and microscopy (Supplementary Figures S3 and S4).

The plates were prepared as described above for the bead-based adhesion assay. HEK cells were detached with 0.25% trypsin and 1 mM EDTA and resuspended at 6 × 10^5^ cells/mL with binding buffer (50 mM Tris, 150 mM NaCl, 1.5% BSA, 2 mM MgCl_2_, 2 mM MnCl_2_, 5 mM d-glucose, pH 7.4). To label the HEK cells with a fluorophore, 1 µg/mL 2′,7′-Bis-(2-Carboxyethyl)-5-(and-6)-Carboxyfluorescein, Acetoxymethyl Ester (BCECF-AM) was added and incubated at 37 °C for 40 min. Cells were diluted to a density of 3 × 10^5^ cells/mL in binding buffer, and EDTA or antibodies were added as described above. After 40 min at 37 °C, 30,000 cells were transferred to each V-well. After incubation for 10 min at room temperature, plates were centrifuged at 200× *g* for 10 min at room temperature with brake off. Non-adherent cells that were accumulated at the bottom of the V-well and were quantified using an Infinite M200 Pro plate reader (Tecan) with excitation at 485 nm and emission at 535 nm. Statistical analysis has been calculated with GraphPad Prism 10.2.0, with an unpaired *t*-test from mean and standard deviation.

## 3. Results

### 3.1. The αI Domains of β_2_ Integrins Can Be Uniformly Expressed in E. coli

**Figure 1:**
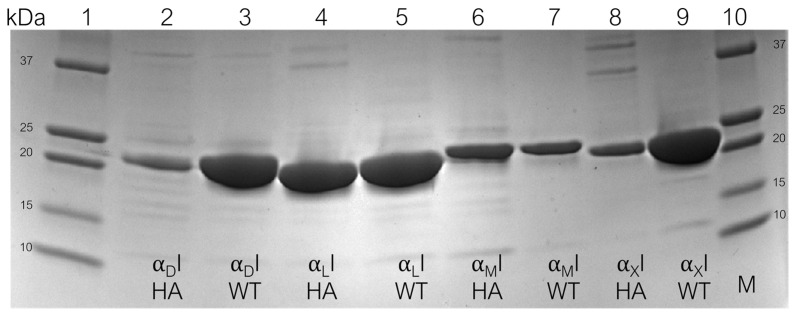
SDS-PAGE analysis of purified recombinant αI domains of four β_2_ integrins in their wild-type (WT) and high-affinity (HA) variants. The gel visualized after Coomassie blue staining. Molecular weight marker (lanes 1, 10), α_D_I (lanes 2, 3), α_L_I (lanes 4, 5), α_M_I (lanes 6, 7), and α_X_I (lanes 8, 9). The image shows His-fused proteins under non-reducing conditions. The original uncropped image can be found in the Supplementary Material as Figure S5.

To achieve a comparable assay system, the expression of all αI domains in the same expression system and uniform processing is critical. Based on several reports on the successful production of individual domains published by us and other groups [42,47], and to generate high yield and facilitate mutagenesis, we selected a prokaryotic expression system (*E. coli* strain BL21(DE3)). The cDNA sequences were chosen to cover the same segment of the corresponding family member’s αI domain (i.e., α_M_I Q146–A334, α_X_I Q148–I336, α_D_I P145–A334, α_L_I G153–Y334, Supplementary Table S1). For each receptor, plasmids encoding the WT and the HA variant were constructed. Most of the HA-inducing mutations had previously been reported, including α_M_I p.I332G [38] and α_X_I p.I333G [14]. In α_L_I, two mutations (K312C/K319C) are used to stabilize the HA form, resulting in an additional disulfide bridge [44]. In the case of α_D_I, similar mutations, (i.e., K287C/K294C) have been reported to generate an HA I domain [18]. However, considering the substantial sequence homology between α_D_I and α_M_I/α_X_I, in contrast to α_L_I, we hypothesized that an isoleucine-to-glycine mutation of the corresponding residue, i.e., α_D_I p.I332G, would result in a HA variant by destabilizing the closed conformation that is usually maintained by isoleucine fitting into a hydrophobic pocket, as reported for α_M_I and α_X_I [38]. Besides a screening of colonies to identify the highest-yielding clones for scale-up, the expression parameters have been optimized to lower temperature (18 °C) for an extended period (65 h). These steps were found critical to achieve soluble protein with high yield. After purification, SDS-PAGE revealed single protein bands for each protein under reducing and non-reducing conditions, with an apparent molecular weight corresponding to the reported and/or calculated value of the corresponding αI domain (Figure 1). To validate proper folding, we determined a melting curve by nanoDSF (Supplementary Figure S2) and validated the correct conformation by performing a direct binding assay (SPR) with the literature-reported ligands, such as ICAM-1 and iC3b. Using a uniform and optimized protein design, expression, and purification platform, all αI domains were produced as WT and HA form at high purity and yield. Interestingly, the disulfide-stabilized HA α_L_I showed the highest yield among the HA forms, which might be explained by the stabilization of the HA conformation by a covalent disulfide bond, while the Ile-to- Gly, mutations lead to a destabilization of the closed conformation, which might result in reduced levels of expressed protein.

### 3.2. β_2_-Integrin I Domains Show Distinct Binding to C3-Derived Opsonins

We established an SPR assay to determine affinity and kinetic parameters of the literature-reported ligands binding to the recombinant αI domains in HA and WT form. Due to the role of CR3 and CR4 in complement-mediated phagocytosis, the α_M_I and α_X_I domains recognize C3-derived opsonins, with iC3b beeing reported as the main ligand [12,15,48]. To mimic the natural binding mode, C3b was deposited on the sensor chip via its thioester moiety upon convertase mediated C3 activation in a quasi-physiological manner (Supplementary Figure S6A). Subsequently, plasma-purified enzymes (factor I) and cofactors (FH, CR1) were used to convert C3b to iC3b and C3dg, respectively (Supplementary Figure S6A). The C3 degradation products immobilized in quasi-physiological manner showed expected binding behavior to their ligands, e.g., factor B to C3b (Supplementary Figure S6B). The WT and HA αI domains of all four β_2_ integrins were injected over the C3b, iC3b, and C3dg surfaces in buffer containing Mg^2+^ to reflect physiological conditions and to maintain MIDAS’ activity. Additionally, a combination of Mg^2+^ and Mn^2+^ was used to induce/stabilize high-affinity conformations of the WT αI domains [49] or EDTA to prevent metal ion-dependent interactions.

**Figure 2:**
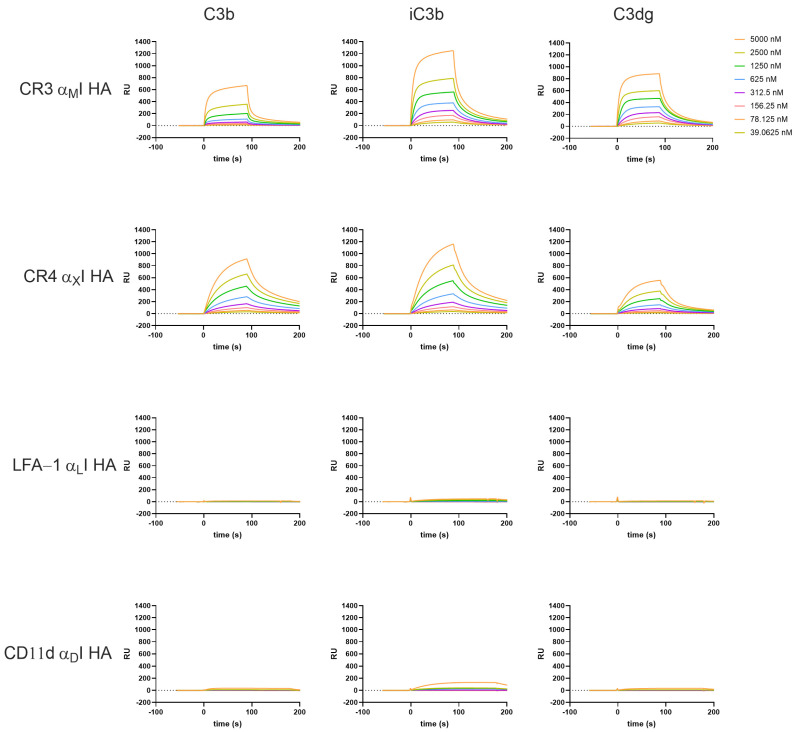
Ligand interaction profile of the high-affinity (HA) variants of recombinant β_2_-integrin αI domains for C3-derived opsonins as measured by SPR in Mg^2+^-containing buffer. The assay was performed as described in Section 2.

The recombinant HA α_M_I showed binding to iC3b and C3dg, which is in agreement with previous reports [11,13,42,50,51], and to a lesser extent, binding to C3b (Figure 2 top row). Interestingly, CR4 HA α_X_I not only bound to C3b and iC3b, as expected [11], but also showed notable binding to C3dg (Figure 2 second row), which had not been investigated before. Furthermore, in direct comparison to HA α_M_I, it becomes apparent that binding kinetics of HA α_X_I to the C3-opsonins showed slower on- and off-rates (Supplementary Figures S7–S10). Performing the assay in EDTA-containing buffer completely abrogated the binding, thereby confirming specific binding via the MIDAS (Supplementary Figures S7–S10).

The α_L_I domain did not exert affinity for any of the C3 fragments (Figure 2 third row), in line with reports that LFA-1 does not serve as a receptor for complement opsonins [5,52]. Very weak binding was observed in the case of α_D_I for iC3b (Figure 2 bottom row), similar to that reported in a study by Ustinov et al. [53].

**Figure 3:**
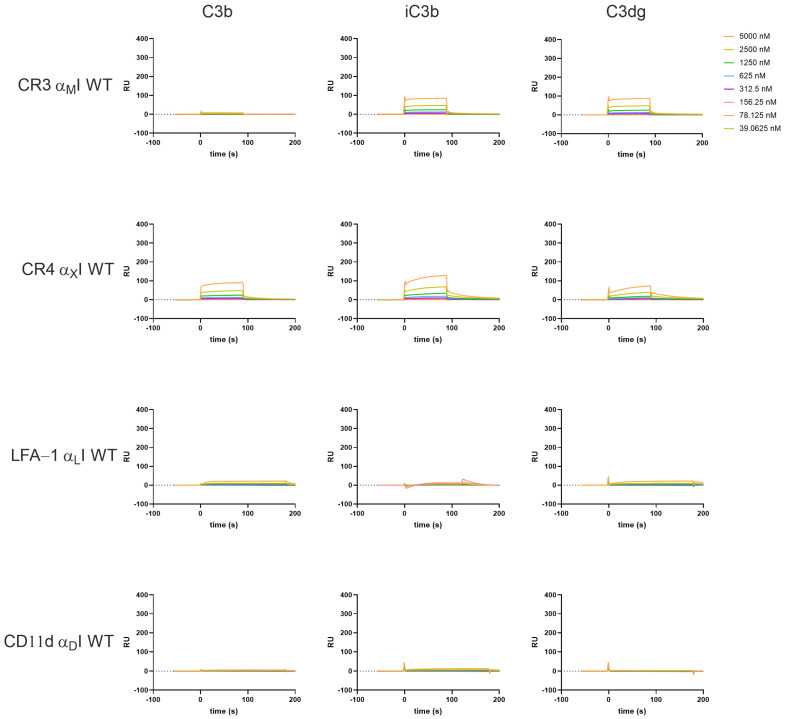
Ligand interaction profile of the wild-type (WT) form of recombinant β_2_-integrin αI domains for C3-derived opsonins as measured by SPR in Mg^2+^ containing buffer. The assay was performed as described in the Section 2.

In line with previous reports, the WT αI domains show much less binding towards the C3-derived opsonins and clear differences regarding binding intensity and kinetics are observed. The overall trend of affinities seen in the HA I domains is maintained with the WT domains; strongest binding was observed with iC3b for α_M_I, and to a lesser extent C3dg, while weak binding interactions are completely abolished in the WT domains (e.g., α_M_I to C3b, Figure 3). In the case of the WT αI domains, we tested the influence of Mn^2+^, as it has been reported to induce high-affinity binding. The addition of Mn^2+^ to the buffer did not substantially influence the binding profiles of the αI WT domains for complement opsonins (Supplementary Figures S7–S10), as Mn^2+^ was reported to stabilize the high-affinity conformation of the full headpiece via AMINDAS and I-like MIDAS. In contrast, adding Mn^2+^ to the HA domains had a notable influence on the kinetic behavior, with α_X_I HA in particular showing faster dissociation from the opsonins (Supplementary Figures S7 and S10).

Adding EDTA abolished binding in most of the cases (Supplementary Figures S7–S14), underlining the important role of divalent cations for the ligand binding. An interesting exemption is α_D_I, as binding signals were still observable for the HA variant binding to iC3b in EDTA buffer (Supplementary Figure S13). Of note, in our beads- and cell-based adhesion assay, we could observe a similar behavior. It remains to be investigated whether α_D_I HA may bind to iC3b in a metal ion-independent manner or whether these results may be technical artifacts.

The obtained binding curves deviate from a Langmuir 1:1 binding model, indicating that there are multiple interaction sites and conformational changes involved, which has also been suggested in the literature [50,54,55]. By fitting the data with a global kinetic rate constant, the SPR analysis allowed for a quantitative comparison of the αI domains binding to the C3-derived opsonins (Table 1). Some of the calculated binding affinities have to be interpreted with caution, as the binding curves did not reach a steady state, while this is assumed by the fitting model, leading to overestimation of the binding affinity. That is especially true for the WT forms, which show a two-state decay. In the case of CR3 α_M_I, the WT form binds weakly yet similarly well to both iC3b and C3dg. Also, the HA state results in a similar selectivity pattern regarding K_D_ values, although iC3b shows ~2-foldhigher SPR responses when compared to C3dg (Table 1, Figure 2). The binding of α_M_I to C3b is detectable in the HA variant, with a 10-fold weaker affinity compared to iC3b and C3dg (K_D_ ∼ 12 µM; Table 1). In contrast, a broader affinity range can be observed for the WT forms of α_X_I (K_D_ ∼ 30–60 µM); it becomes narrower in the HA variant, albeit at a much stronger affinity (K_D_ ~ 2–4 µM; Table 1; full kinetic profiles Supplementary Table S2).

### 3.3. Recombinant αI Domains of β_2_ Integrins Recognize ICAMs

**Figure 4:**
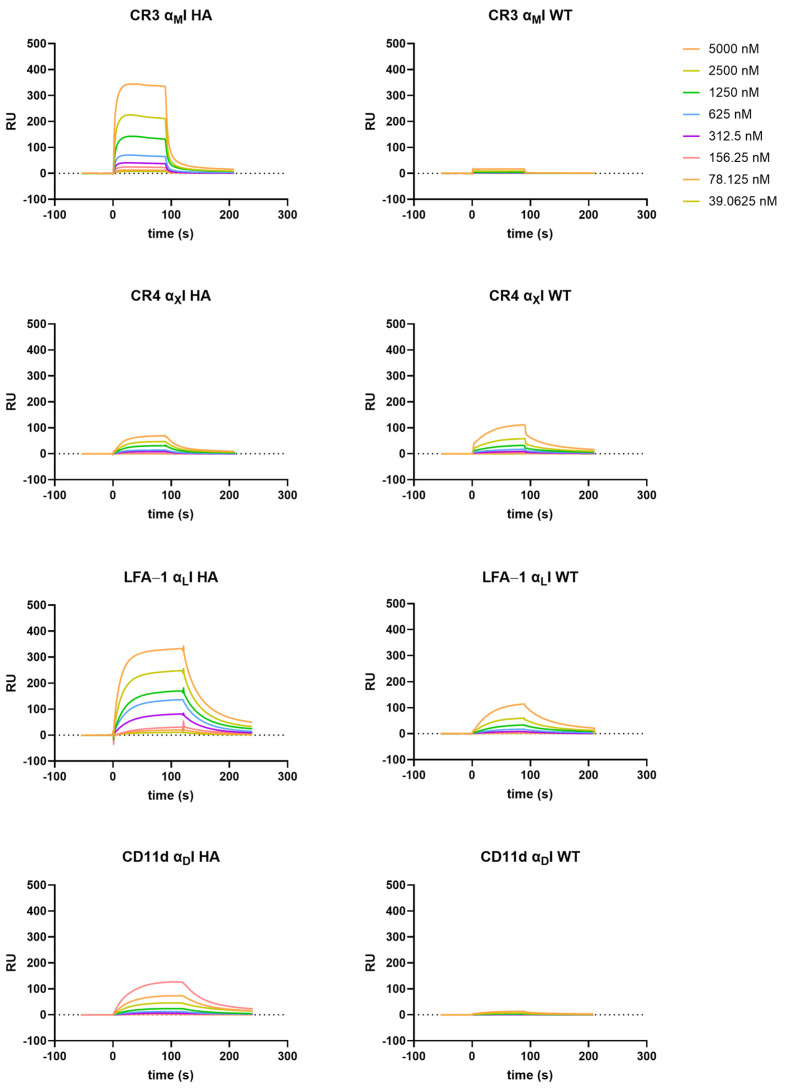
SPR sensorgrams of the β_2_-integrin αI-domain high-affinity (HA) and wild-type (WT) variants binding to immobilized ICAM-1 in MgCl_2_ supplemented HBST. The assay was performed as described in the Section 2.

As ICAMs are reported ligands for all members of the β_2_-integrin family, we tested the full panel of αI domains in a SPR-based binding assay against immobilized ICAM-1, -2, and -3 (Figure 4 and Supplementary Figures S15–S22). Similar as observed with C3-derived opsonins, the αI domains in the WT form showed weak binding to the ICAMs in the case of LFA-1 and CR4 (0.04 µM–5.0 µM; Figure 4 and Supplementary Figures S18 and S20), while for the other WT I domains, no binding is measurable. For all WT αI domains, ICAM-1 showed the highest binding affinity compared to ICAM-2 and -3. This is also true when we tested the HA variants of the I domains, where α_L_I and α_M_I showed particularly strong binding (Figure 4 and Supplementary Figures S15 and S19). Interestingly, in the case of α_X_I, there is no clear difference between the WT and HA variant, with a comparatively low binding affinity even for the HA form (Figure 4 and Supplementary Figures S17 and S18). Similar to ICAM-1, α_L_I and α_M_I HA domains show the strongest binding among the β_2_-αI domains to ICAM-2, while ICAM-3 shows only residual binding. By supplementing Mn^2+^ to the buffer, especially in the case of the HA α_L_I-domain, we observe enhanced binding affinity and kinetics, while the WT α_L_I domain shows no effect. This indicates that Mn^2+^ is not inducing an HA-conformation by itself but it seems to increase binding behavior in the HA form, thereby suggesting that Mn^2+^ induces a preferred binding interface via the MIDAS towards the ICAMs. Of note, in our assay setup, we observed the highest binding signal for α_D_I HA in the case of ICAM-1, even if it had been reported to be a receptor for ICAM-3 [18]. Remarkably, when measuring the I domains in EDTA buffer, only α_D_I HA retained substantial binding affinity (Supplementary Figure S20), as we had it also observed for the C3-derived opsonin iC3b. This observation suggests that ligand binding is not mainly mediated by the MIDAS in CD11d/CD18. Interestingly, the binding of ICAM-1 to the α_L_I domain of LFA-1 was also not fully abolished in EDTA buffer.

### 3.4. The Interaction Platform Enables the Characterization of β_2_-Integrin Modulators

**Figure 5:**
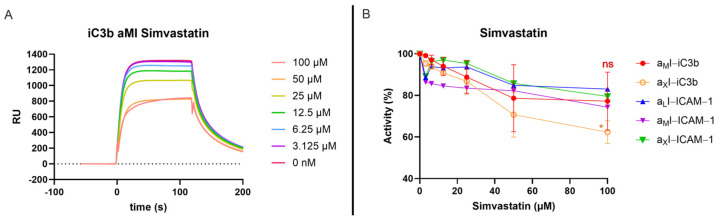
Interference of simvastatin in ligand binding to β_2_-integrin αI domains measured by a competitive SPR assay. The αI domains were preincubated at a fixed concentration of 5 µM with a dilution series of simvastatin (3–100 µM) in HBST supplemented with 1 mM MgCl_2_ and 5% DMSO. (**A**) Representative sensorgram of CR3 α_M_I binding to iC3b in absence and presence of simvastatin. (**B**) Concentration-dependent inhibition of ligand binding by simvastatin for all three tested αI domains, shown as percentage of non-inhibited binding signal, as mean and standard deviation; *p* ≥ 0.05 = ns, 0.01 > *p* > 0.05 = *.

Alongside the elucidation of the β_2_-integrin ligands and their specificity within the β_2_ family, the SPR assay has been developed to determine the affinity of integrin modulators, targeting the αI domains. As a proof-of-concept for such an application, we investigated the binding of simvastatin to the β_2_-integrin αI domains. Simvastatin had been described to inhibit ligand recognition of LFA-1 at higher concentrations [56,57]. Furthermore, structural and functional studies proposed that simvastatin could serve as a competitive antagonist at α_M_I via binding to the MIDAS [58]. Therefore, we tested the antagonistic properties of simvastatin against α_M_I, α_X_I, and α_L_I in a competitive SPR assay format. For this purpose, ICAM-1 and iC3b were immobilized on a sensor chip as described above. All αI domains were preincubated at a fixed concentration (5 µM) with a dilution series of simvastatin (3 µM–100 µM) before injection onto the ligand surfaces. Although simvastatin was able to partially inhibit the binding of α_M_I and α_X_I to iC3b in a dose-dependent manner, no complete inhibition could be achieved (residual activities of 77% and 63%, respectively, Figure 5 and Supplementary Figure S23). This result is in line with reports of multiple binding interactions of iC3b to the α_M_I domain, which might account for residual binding after the MIDAS interaction is blocked. In contrast, binding of all three αI domains to ICAM-1 was inhibited only weakly, with residual binding activities of 83% (α_L_I), 80% (α_X_I), and 74% (α_M_I) (Figure 5 and Supplementary Figure S23), which is similar to the first reports, that simvastatin is not competing with α_M_I binding to ICAM-1 [58]. Furthermore, this is indicating that the MIDAS cannot be the only interaction site for ICAM-1 with the β_2_ integrins, as our results of the residual binding of α_L_I to ICAM-1 in EDTA also suggested.

### 3.5. The Recombinant αI Domains Can Be Used in Functional Adhesion Assays

**Figure 6:**
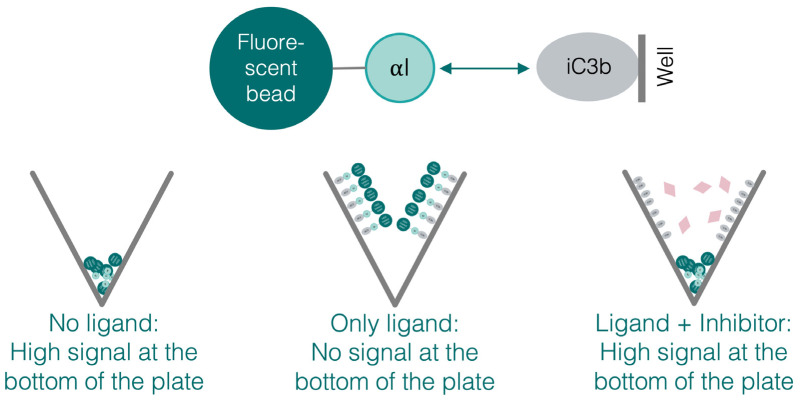
Schematic presentation of the V-well adhesion assay.

The adhesion of leukocytes to cells and particles during extravasation or phagocytosis marks the central functional feature of β_2_-integrin receptors. Whereas the full receptor heterodimer is involved in the resulting effector mechanism, the main binding site for the ligand is the αI domain. To validate the functionality of the recombinant domains and compare them with the corresponding full receptor, we established two adhesion assays. One assay is using the isolated, recombinant αI domains coated on fluorescent beads; the other assay is using HEK293 cells stably transfected with β_2_ integrins (CR3, CR4, and CD11d/CD18; a corresponding LFA-1 cell line could not be successfully generated). V-shaped wells in a 96-well format were coated with ligands specific for each αI domain, i.e., iC3b for CR3 and CR4 and ICAM-1 for LFA-1 and CD11d/CD18. In case of an αI-domain-mediated adhesion to the ligands iC3b or ICAM-1, beads or cells are expected to adhere to the ligand-coated wall, whereas unbound beads and cells accumulate at the bottom of the plate after centrifugation (Figure 6). Within this setup, the assay allows to compare adhesion to endogenous ligands, the influence of modulators on adhesion, and the role of the divalent cations. Using a plate reader with a narrow detection geometry, the amount of fluorescently labeled cells or beads accumulating at the bottom of the plate can be quantified and compared to the fluorescence of the full well. The main advantage of this assay setup is that the washing steps required for adhesion assays in standard flat-bottom plates can be omitted, and that non-adherent cells accumulate at the bottom of the well after centrifugation [59]. In the bead-based assay, an anti-6xHis mAb was used to capture the αI domains in their HA variant on the surface of fluorescent beads in an oriented manner via their purification tag. Although CR3 is known to adhere to uncoated polystyrene [60], this phenomenon was not observed under our experimental conditions.

**Figure 7:**
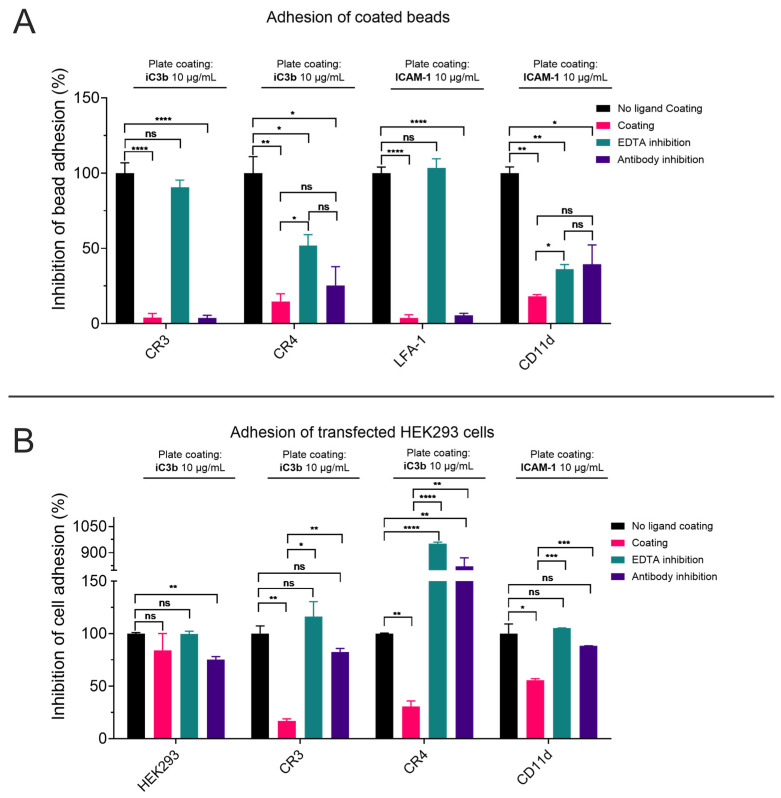
Adhesion assays based on αI domain-coated beads (**A**) or β_2_-integrin-transfected HEK293 cells (**B**). iC3b-coated wells were used to measure CR3- and CR4-mediated adhesion, and ICAM-1-coated wells for LFA-1 and CD11d-/CD18-mediated adhesion. Inhibition by EDTA or domain-specific blocking antibodies was determined in comparison to non-ligand coated wells. Statistical analysis has been calculated with GraphPad Prism 10.2.0, with an unpaired *t*-test from mean and standard deviation, with *p* ≥ 0.05 = ns, 0.01 > *p* > 0.05 = *, 0.001 > *p* > 0.01 = **, 0.0001 > *p* > 0.001 = *** and <0.0001 = ****.

As expected, α_M_I- and α_X_I-coated beads adhered to iC3b, whereas α_L_I- and α_D_I-coated beads adhered to ICAM-1. When adding EDTA to the buffer, the adhesion was completely abolished for the αI domains of CR3 and LFA-1, while only a partial inhibition was observed for the CR4 and CD11d/CD18 αI domains, similarly to what was observed in the SPR assay in the case of α_D_I. We also tested the ability of blocking antibodies to inhibit αI-domain-mediated adhesion to the immobilized ligands. Especially in the case of an anti-CD11a antibody with reported binding epitope on the I domain a full inhibition of adherence was observed. Similarly, the anti-CD11b mAb M1/70 has a strong impact on adhesion, whereas the antibodies for CD11c and CD11d used in this study only showed partial inhibition of adhesion (Figure 7A), even if at least anti-CD11c mAb 3.9 has been described as I-domain-binding antibody [61].

To compare the adhesion properties of the recombinant HA αI domains with those of the corresponding full receptors, a similar assay was performed with HEK293 cells that were stably transfected with CR3, CR4, or CD11d/CD18 [18]. After verifying by flow cytometry that the cells expressed the individual β_2_-integrin receptors (Supplementary Figure S3), the cells were fluorescently labeled with BCECF-AM and incubated in ligand-coated wells in analogy to the bead-based assay. CR3- and CR4-transfected HEK293 cells both adhered to iC3b-coated wells, while CD11d-/CD18-transfected cells bound to ICAM-1, confirming the results obtained by SPR. To verify that the adherence of the HEK293 cell was dependent on the level of overexpressed integrins, we tested wild-type HEK293 cells in parallel; as expected, a variation of assay conditions had little effect on adhesion properties of non-transfected cells (Figure 7B). In contrast to using the recombinant αI domains on beads, EDTA could fully inhibit adhesion in case of all full integrins, which is in line with the reported role of the I-like MIDAS and AMINDAS in the β_2_ chain of the integrins. Similarly, domain-specific antibodies were able to inhibit adhesion on the full integrin much better when compared to αI domain-coated beads (Figure 7B). This is of no surprise, as for the most used antibodies, the epitope is located somewhere on the α-chain at a site available in the full integrin but not necessary in the αI domain.

Of note, the CR4-transfected HEK293 cells also adhered to uncoated control wells. Nevertheless, a difference in signal of CR4-transfected HEK293 cells binding to uncoated plastic and to coated iC3b was still observable. Since the value obtained from the uncoated wells was set to 100% as the reference point, the values for the inhibition of adhesion by EDTA and the anti-CD11c antibody resulted in values higher than 100%.

## 4. Discussion

In this study, we reported the recombinant expression of the ligand-binding αI domains of the β_2_-integrin family using the same constructs, to enable a direct comparison and molecular characterization of ligand binding within the β_2_-integrin family. We employed the recombinant αI domains in different applications ranging from molecular interaction analysis (SPR) to functional studies (adhesion assay) and validated our assay platform by assessing ligands and functional properties reported in literature.

By utilizing standardized protein production and assay conditions, our study enabled the first direct comparison of binding behavior via the αI domain for all members of the β_2_-integrin family, in their WT and HA variants. For a long time, it has been postulated that, on resting cells, the αI domain is present in the closed conformation (in our platform WT) and displays very weak to non-detectable affinity toward its ligands [14]. We validated this observation by SPR, where the WT αI domains indeed showed no or weak interaction with their reported ligands iC3b and ICAM-1. It has been postulated that upon inside-out activation, structural changes of the entire integrin towards an extended conformation also affect the αI domain, which is reported to adapt a high-affinity state [3]. To induce the HA conformation of an isolated αI domain, mutagenesis can be applied; it has been reported that the closed conformation is stabilized when an Ile residue is bound in a binding pocket referred to as “socket for isoleucine” (SILEN). By mutating this isoleucine to a glycine residue, the equilibrium between the closed and open conformation is shifted to the open HA state [14,38]. In the case of α_L_I and α_D_I, another strategy has been reported, where an additional disulfide bridge was introduced to lock the αI domain in the active conformation [18,44]. As CD11d shows high sequence homology with CD11b and CD11c, including the conserved isoleucine binding to the SILEN, we rationalized that the p.I332G mutation in α_D_I would induce the HA form. Indeed, our hypothesis was supported by results from the SPR assay, where α_D_I HA showed substantially improved ligand binding, for example, to ICAM-1. CD11d/CD18 is the least investigated β_2_ integrin with scarce information on endogenous ligands. Among those, binding to ICAM-3 and vascular cell adhesion molecule 1 (VCAM-1) are documented best [19,20]. To our knowledge, no direct interaction between CD11d/CD18 and ICAM-1 has been reported before. Furthermore, given its high sequence similarity to CR3 and CR4, it is surprising that CD11d/CD18 has never been reported as receptor for C3-derived opsonins, especially when considering that CD11d/CD18 shares many ligands with CR3, for example, CCN1 and fibrinogen [62,63]. While the α_D_I HA domain showed weak binding to iC3b in our SPR assay, it remains to be further investigated whether this is a relevant interaction also observable for the full-length CD11d/CD18 or based on an assay artefact. In this context, it is interesting to note that EDTA was not able to diminish binding of α_D_I to neither ICAM-1 nor iC3b, which might indicate that the interaction of the α_D_I domain to these ligands does not primarily occur via the MIDAS. This observation from our SPR assay is at least in line with the bead-based adhesion assay using the purified α_D_I domain, where EDTA could also not fully abolish binding to ICAM-1. Furthermore, the initial reporting of the ICAM-3 binding to CD11d did not investigate the dependence on divalent cations [20]. Quite notable is a recent publication highlighting a role of a heterodimer between α_D_ and β_1_ in colorectal cancer, where it seems to mediate localized oxygen scavenging by binding hemoglobin, resulting in growth advantages for colorectal cancer in hypoxic environments [64].

We were able to detect binding of the high-affinity CR3 α_M_I domain to C3b, which showed dependence on divalent cations. However, the literature reports on C3b binding of CR3 are scarce, as mostly iC3b is investigated as the binding partner, and the few reports on direct binding are inconsistent. While Bajic et al. showed binding of both, CR3 and CR4 αI domains in their HA form to C3b [11], Lin et al. did show no binding of the HA α_M_I to C3b [42]. A reason for the different results might lie in the differences of used constructs, expression systems (e.g., *E. coli* vs. yeast [42]), and surface density of the immobilized C3b. There are multiple studies, which indirectly support our observation of α_M_I binding to C3b: Xu et al. reported that α_M_I binds to the TED domain of iC3b and C3dg, which is hidden and not reachable for ligand binding in the C3b fragment but becomes accessible through cleavage of C3b by FI and FH at the C-terminal part of the CUB domain [55]. A recent study by Fernandez et al. reported a crystal structure of α_M_I HA (p.C128S/I316G) in complex with iC3b, and confirmed that the αI domain can bind to iC3b via its TED domain. Furthermore, depending on the conformation of the α_M_I-iC3b complex, the αI domain can also bind to a site between MG1-MG2 and MG6-MG7 on the C3c moiety [50]. This crystallographic insight is in line with our SPR results., as binding areas of α_M_I on MG6-MG7 and MG1-MG2 are accessible for binding, especially when iC3b and C3b are immobilized on the chip surface in high density. iC3b shows the highest binding affinity among all opsonins, since multiple binding sites are involved (C3c and TED domain). However, based on these results, it also becomes evident C3b is unlikely a physiologically relevant CR3 ligand as access to the TED domain is sterically blocked by the CUB domain for the full-length integrin [50].

CR4 is reported to bind to iC3b via a binding site on MG3-MG4 on the C3c moiety, which has been investigated by Chen et al. using the WT ectodomain of CR4 for their experiments [54]. The analogous binding site is postulated to also be accessible in C3b [54]. Furthermore, CR4 binds via a second binding site, i.e., the C345c knob on the C3c moiety, to iC3b [55]. Cleavage of C3b to iC3b does not lead to a different exposure of MG3 and MG4, but to a distinct orientation of the C3c domain. This might explain CR4’s higher binding response for iC3b over C3b that we also could detect in the study presented here. Moreover, we detected binding of HA α_X_I to C3dg in our SPR assay, which confirms previous observations from Bajic et al. [11]. As earlier reports revealed that CR3 and CR4 do not have overlapping binding sites on iC3b [55], concurrent binding of both receptors to iC3b could be possible and lead to improved recognition of opsonized particles.

As described before, integrins show the highest affinity to their ligands when the αI domain assumes an “open” conformation, which is mimicked with the HA αI domains in our assay platform. Although the WT αI domains, which assume a “closed” conformation, also showed binding to their ligands in our SPR studies, the response intensity was much lower and binding kinetics showed a biphasic decay (see Figure 3, Figures S8, S10 and S18). This is in line with previous observations that ligand binding to the αI domains is conformationally regulated, with a downward movement of the C-terminal helix (α7) that leads to integrin activation. The biphasic decay indicates the existence of a secondary conformational transition, which is induced by the primary ligand–I-domain complex and shows a higher complex stability, resulting in a slower dissociation-rate. To analyze the multistep binding, sophisticated computational models have been established to fit the kinetic profile [65] and simulate the complex transition states [66].

With our SPR analysis, we were able to demonstrate the inhibitory effect of modulators in a competitive binding assay format. As a proof-of-concept study, we evaluated simvastatin as a known inhibitor of LFA-1 and CR3 [56,58]. The inhibition of ICAM-1 binding to both α_L_I and α_M_I by simvastatin was rather weak and incomplete. Of note, previous studies were not able to show the inhibition of ICAM-1 binding to CR3 when testing simvastatin on the HA α_M_I domain [58]. While we did detect decreased binding of CR3 and CR4 to iC3b in the presence of simvastatin, again, no complete inhibition was achieved. Furthermore, it must be noted that the competitive setup with the weak inhibitor simvastatin resulted in high standard deviations, omitting statistical significance for most examples and tested concentrations. At least for CR3, there is structural evidence of an antagonistic binding mode of simvastatin, which is complexing the Mg^2+^ of the MIDAS, albeit with low affinity [58]. Given the sequence similarity of CR4 with CR3, it is not surprising that the CR4–iC3b binding was also partially and statistically significantly inhibited. In principle, an incomplete impairment of ligand binding likely excludes a full antagonistic behavior of simvastatin. It suggests a partial antagonism via low affinity or an adjacent site or an allosteric modulation through conformational changes as possible modes. At least in the case of LFA-1, an allosteric mechanism has been reported for statins [56].

Comparing the results obtained from both adhesion assays and SPR, both CR3 and LFA-1 exhibit particularly strong binding to the corresponding ligand, which can be completely inhibited with EDTA. This underlines the importance of the isolated αI domain for ligand binding and that the divalent cation in the MIDAS plays an important role for the interaction. Nevertheless, it also became evident that in using isolated αI domains, not all relevant interactions can be observed. The role of additional binding sites on the α-chain, and the role of divalent cations in the conformational stability of the full-length integrin, especially underlines the necessity to validate results obtained with isolated αI domains using assays involving the full integrin. While not positioned to fully address complex interaction and functional aspects, the molecular interaction assays (SPR and beads-based assay) nevertheless provide a valuable tool to identify or exclude binding areas and binding modes of the αI domain involved in ligand interaction. An example within this study is α_D_I binding to ICAM-1, which does not seem to be dependent on the MIDAS.

Interestingly, no mAb was able to fully inhibit binding of the αI domains to their respective immobilized ligands in the bead-based adhesion assay, even if these antibodies had been described as blocking antibodies in earlier studies [61,67,68]. Notably, most of these studies used the mAbs on cells expressing the full CD18 integrins; therefore, the characterization of the αI domain as binding epitope might need reconsideration. This is also underlined by results of the cell-based adhesion assay presented here, where the reported antibodies were able to antagonize adhesion via the full-length receptors.

One major limitation of our platform is the use of isolated αI domains for the molecular interaction studies. Overall, the I domains are recognized as major ligand-binding domains and thereby constitute a suitable for modulators, which provides the rationale of our approach. Nevertheless, there are multiple reports, especially for iC3b, which show the involvement of other domains in binding, such as the β-propeller. Additionally, by using isolated αI domains in our SPR assay, we expose binding surfaces that might be shielded in the integrin heterodimer. This is likely the reason, why we observe a notable interaction between α_M_I and C3b, even if C3b is not a reported physiological ligand for CR3. By using the full heterodimeric ectodomain, this limitation could be omitted. Furthermore, we used mutational approaches to generate a HA conformation in the isolated αI domains; this may limit the domain flexibility that is known to be an important aspect of integrin biology, as discussed for the observed biphasic decay of the WT I domains. By using mutational approaches for generating the HA-form, we might generate artificial conformations, which are only inefficiently resembling the “open” conformation. Nevertheless, we were able to validate our approach by reproducing binding behavior with the literature-reported ligands.

## 5. Conclusions

In conclusion, we present an assay platform which, for the first time, compares all four β_2_-integrin family members side by side on a molecular level. The combination of direct binding studies with functional adhesion assays allowed us to confirm previous observations regarding the ligand selectivity of β_2_-integrin and provided new insight, as in the case of CD11d/CD18. It has to be noted that the main application of the newly established platform lies in the elucidation of αI domain-mediated interactions and enable screening approaches for modulators that target this domain. Although the platform may also provide functional insight, it will be critical to validate any results in the context of the full β_2_-integrins. Even for the molecular studies presented here, additional experiments are warranted to further validate the platform. This is particularly important in the case of α_D_I, where confirmed ligands are scarce and the observation of Mg^2+^-independent ligand binding is possible but rather unexpected. Currently, our cell-based adhesion assay is restricted to CR3, CR4, and CD11d/CD18 and does not include LFA-1. Since these three β_2_ integrins share a greater sequence identity when compared to LFA-1, our platform is still valuable to explore comparative ligand binding. In the future, the platform could be extended by producing HEK293 cells stably transfected with LFA-1. Furthermore, it would be desirable to include assays that describe other functional aspects, such as a phagocytosis or transmigration. The recombinant αI domains and assays established here are suitable to characterize the interaction of β_2_ integrins with potential ligands in a quantitative and comparative manner. The platform enables insight into the ligand-binding profiles among β_2_ integrins and allows screening and optimization of target-specific modulators for research and therapy. Even though a general inhibition of integrins for therapeutic purposes is typically considered challenging due to the expected impact on immune surveillance, a tailored blockage of specific integrin–ligand interaction may be beneficial in certain disorders. Importantly, and independent of their therapeutic potential, the availability of integrin/ligand-specific modulators will be of critical importance to further elucidate the intricate functional interplay of integrin receptors in health and disease.

## Figures and Tables

**Table 1:** K_D_ values of αI domain binding to C3 fragments in Mg^2+^ buffer. n.b. = no binding. Displayed is the mean ± SD, with n = 5. * The K_D_ of the WT should be interpreted with caution, as the fits are overestimating the RUmax.

	α_M_I		α_X_I		α _L_I		α_D_I	
	HA [µM]	WT [µM] *	HA [µM]	WT [µM] *	HA	WT	HA	WT
C3b	11.6 ± 9.8	n.b.	2.2 ± 1.5	55.5 ± 27.1	n.b.	n.b.	n.b.	n.b.
iC3b	1.8 ± 1.0	15.0 ± 9.5	2.6 ± 1.6	33.0 ± 17.8	n.b.	n.b.	n.b.	n.b.
C3dg	1.4 ± 0.2	16.1 ± 10.2	3.8 ± 2.4	30.4 ± 12.0	n.b.	n.b.	n.b.	n.b.

## Data Availability

Dataset available on request from the authors. The raw data supporting the conclusions of this article will be made available by the authors on request.

## Supplementary Materials

The following supporting information can be downloaded at https://www.mdpi.com/article/10.3390/biom15020238/s1. Table S1. Overview on used sequences of the recombinant proteins. Table S2. Full kinetic profile of CR3 αMI and CR4 αXI binding to C3-derived opsonins. Figure S1. Sequence alignment of all β2-Integrin αI domains. Figure S2. nanoDSF experiment of recombinant αI-domains in wild type (WT) and high affinity (HA) constructs in comparison to buffer alone. Figure S3. HEK cell lines imaged with confocal fluorescence microscopy at 60x magnification. Figure S4. HEK cells were further characterized using flow cytometry. Figure S5. Uncropped, original gel of gel shown in Figure 1. Figure S6. A: SPR Sensorgram of deposition of C3b and conversion to iC3b and C3dg. B: As a control that C3b was cleaved successfully, single concentrations of different ligands were injected on all flow cells. To test if the cleavage of iC3b with FH and FI and C3dg with CR1 and FI was successful, single concentrations of different ligands were injected on all flow cells. Figure S7. CR3 αMI domain high-affinity binding to C3 fragments. SPR sensorgrams. Figure S8. CR3 αMI domain wild type binding to C3 fragments. SPR sensorgrams. Figure S9. High-affinity CR4 αXI domain binding to C3 fragments. SPR sensorgrams. Figure S10. CR4 αXI domain wild type binding to C3 fragments SPR sensorgrams. Figure S11. LFA-1 αLI high-affinity domain binding to C3 fragments. SPR sensorgrams. Figure S12. LFA-1 αLI wild type domain binding to C3 fragments. SPR sensorgrams. Figure S13. CD11d/CD18 αDI high-affinity domain binding to C3 fragments. SPR sensorgrams. Figure S14. CD11d/CD18 αDI wild type domain binding to C3 fragments. SPR sensorgrams. Figure S15. CR3 αMI domain high-affinity binding to ICAM’s. SPR sensorgrams. Figure S16. CR3 αMI domain wild type binding to ICAM’s. SPR sensorgrams. Figure S17. CR4 αXI high-affinity domain binding to ICAM’s. SPR sensorgrams. Figure S18. CR4 αXI wild type domain binding to ICAM’s. SPR sensorgrams. Figure S19. LFA-1 αLI high-affinity domain binding to ICAM’s. SPR sensorgrams. Supp. Figure S20. LFA-1 αLI wild type domain binding to ICAM’s. SPR sensorgrams. Figure S21. CD11d/CD18 αDI high-affinity domain binding to ICAM’s. SPR sensorgrams. Figure S22. CD11d/CD18 αDI wild type domain binding to ICAM’s. SPR sensorgrams. Figure S23. Competitive SPR using Simvastatin as an antagonist of β2-integrins.

## Abbreviations

The following abbreviations are used in this manuscript:

AMINDAS	adjacent to MIDAS
BCECF-AM	2′,7′-Bis-(2-Carboxyethyl)-5-(and-6)-Carboxyfluorescein, Acetoxymethyl Ester
CR1	Complement receptor 1, CD35
CR3	Complement receptor 3, macrophage-1antigen, αMβ2, CD11b/CD18
CR4	Complement receptor 4, p150,95, αXβ2, CD11c/CD18
ECM	Extracellular matrix
EDTA	Ethylenediaminetetraacetic acid
FB	Complement factor B
FBS	Fetal bovine serum
FD	Complement factor D
FH	Complement Factor H
FI	Complement Factor I
FITC	Fluorescein isothiocyanate
FRET	Förster resonance energy transfer
GST	Glutathione-S-Transferase
HA	High affinity
HEK293	Human Embryonic Kidney 293 cells
HEPES	2-(4-(2-Hydroxyethyl)-1-piperazinyl)-ethansulfuric acid
ICAM	Intercellular adhesion molecule
IPTG	Isopropyl β-D-1-thiogalactopyranoside
LAD	Leucocyte adhesion deficiency
LFA-1	Lymphocyte function-associated antigen 1, αLβ2, CD11a/CD18
MIDAS	Metal ion-dependent adhesion site
nanoDSF	Nano differential scanning fluorimetry
rpm	Revolutions per minute
RU	Response units
SILEN	Socket for isoleucine
SPR	Surface plasmon resonance
VCAM-1	Vascular cell adhesion molecule 1
vWFA	Von Willebrand factor type A
WT	Wild type
