# Supplementary material for: Determining Ligand Binding and Specificity Within the β2-Integrin Family with a Novel Assay Platform
Source: Biomolecules. 2025 Feb 7;15(2):238. doi: 10.3390/biom15020238 (PMC11853025; doi:10.3390/biom15020238)
Supplement: Supplementary file 1 [file biomolecules-15-00238-s001.zip › biomolecules-3454275-supplementary.pdf]

Supporting Information

Determining ligand binding and specificity within the β_2-integrin family with a novel assay platform

Carla Johanna Sommer-Plüss[1], Céline Leiggener[1], Elira Nikci[1], Riccardo Vincenzo Mancuso[1], Said Rabbani[1], Christina Lamers[1,2]*, and Daniel Ricklin[1]*

[1] Molecular Pharmacy Research Group, Department of Pharmaceutical Sciences, University of Basel, Klingelbergstrasse 50, 4056 Basel, Switzerland

[2] Institute for Drug Development, Faculty of Medicine, University of Leipzig, Brüderstraße 34, 04103 Leipzig

Correspondence*:

Prof. Dr. Daniel Ricklin, d.ricklin@unibas.ch

Jun.-Prof. Dr. Christina Lamers, christina.lamers@uni-leipzig.de

Table of content

Table S1. Overview on used sequences of the recombinant proteins. The αI-domains were produced for each β_2-integrin in a wild type and a high-affinity variant.

Uniprot Entry	Protein	Sequence	HA mutation	Reference
Q13349	α_DI	P145 – A334	I332G	(1)
P20701	α_LI	G153 – Y334	K312C/K319C	(2)
P11215	α_MI	Q146 – A334	I332G	(3)
P20702	α_XI	Q148 – I336	I333G	(4)

Table S2. Full kinetic profile of CR3 α_MI and CR4 α_XI binding to C3-derived opsonins as mean ± SD, with n = 5.

	α_MI				α_XI			
	HA		WT		HA		WT	
	k_a (10^4/Ms)	k_d (10^{-2}/s)	k_a (10^4/Ms)	k_d (10^{-2}/s)	k_a (10^4/Ms)	k_d (10^{-2}/s)	k_a (10^4/Ms)	k_d (10^{-2}/s)
C3b	1.5 ± 1.0	14.3 ± 6.6	-	-	1.4 ± 0.7	4.0 ± 4.4	0.9 ± 0.4	49.3 ± 35.0
iC3b	2.7 ± 0.5	4.6 ± 1.9	632 ± 884	6620 ± 8515	1.3 ± 0.5	3.3 ± 1.8	0.3 ± 0.0	10.9 ± 7.2
C3dg	3.5 ± 1.2	4.9 ± 1.5	83.4 ± 80.1	1478 ± 1122	0.9 ± 0.4	2.9 ± 1.2	0.2 ± 0.0	5.1 ± 2.4

```
alphaL      --GNVDLVFLFDGSMSLQPDEFQKILDFMKDVMKKLSNTSYQFAAVQFSTSYKTEFDFSD        58
alphaX      ASQEQDIVFLIDGSGSISSRNFATMMNFVRAVISQFQRPSTQFSLMQFSNKFQTHFTFEE        60
alphaM      -QEDSDIAFLIDGSGSIIPHDFRRMKEFVSTVMEQLKKSKTLFSLMQYSEEFRIHFTFKE        59
alphaD      PHQEMDIVFLIDGSGSIDQNDFNQMKGFVQAVMGQFEGTDTLFALMQYSNLLKIHFTFTQ        60
               : *:.**:*** *:   :*  :  *:  *: ::.  .  *: :*:*   : .* * :

alphaL      YVKWKDPDALLKHVKHMLLLTNTFGAINYVATEVFREELGARPDATKVLIIITDGEATDS        118
alphaX      FRRSSNPLSLLASVHQLQGFTYTATAIQNVVHRLFHASYGARRDATKILIVITDGKKEGD        120
alphaM      FQNNPNPRSLVKPITQLLGRTHTATGIRKVVRELFNITNGARKNAFKILVVITDGEKFGD        119
alphaD      FRTSPSQQSLVDPIVQLKGLTFTATGILTVVTQLFHHKNGARKSAKKILIVITDGQKYKD        120
            :    .  :*:  : ::   * *  .*  *. .:*.   *** .* *:*::****:   .

alphaL      -------GNIDAAKDIIRYIIGIGKHFQTKESQETLHKFASKPASEFVKILDTFEKLKDL        171
alphaX      SLDYKDVIPMADAAGIIRYAIGVGLAFQNRNSWKELNDIASKPSQEHIFKVEDFDALKDI        180
alphaM      PLGYEDVIPEADREGVIRYVIGVGDAFRSEKSRQELNTIASKPPRDHVFQVNNFEALKTI        179
alphaD      PLEYSDVIPQAEKAGIIRYAIGVGHAFQGPTARQELNTISSAPPQDHVFKVDNFAALGSI        180
                          .:*** **:*  *:   : : *: ::* *  :.:  :: *  *  :

alphaL      FTELQKKIYVI 182
alphaX      QNQLKEKIFAI 191
alphaM      QNQLREKIFA- 189
alphaD      QKQLQEKIYA- 190
             .:*::**:.
```

Figure S1 Sequence alignment of all β_2-Integrin αI domains. *Red* indicates small and hydrophobic amino acids. *Blue* indicates acidic amino acids. *Magenta* indicates basic amino acids. *Green* indicates hydroxyl, sulfhydryl, and amine residues and glycine. * indicates single, fully conserved residues. *:* indicates groups of strongly similar properties. . indicates groups of weakly similar properties. Including α-helix and β-sheet domains.

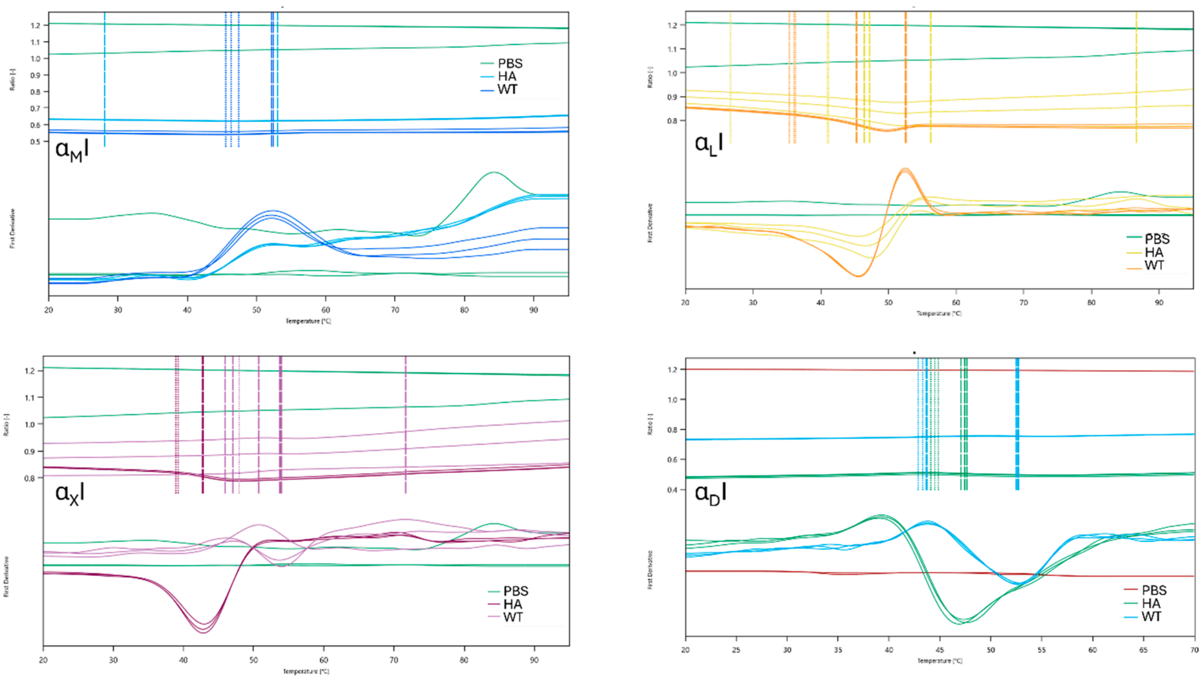

Figure S2 nanoDSF experiment of recombinant αI-domains in wild type (WT) and high affinity (HA) constructs in comparison to buffer alone. Each construct was measured with three replicates. To analyse the melting temperature of the proteins, the ratio of 330 nm and 350 nm and its first derivative have been used and displayed here.

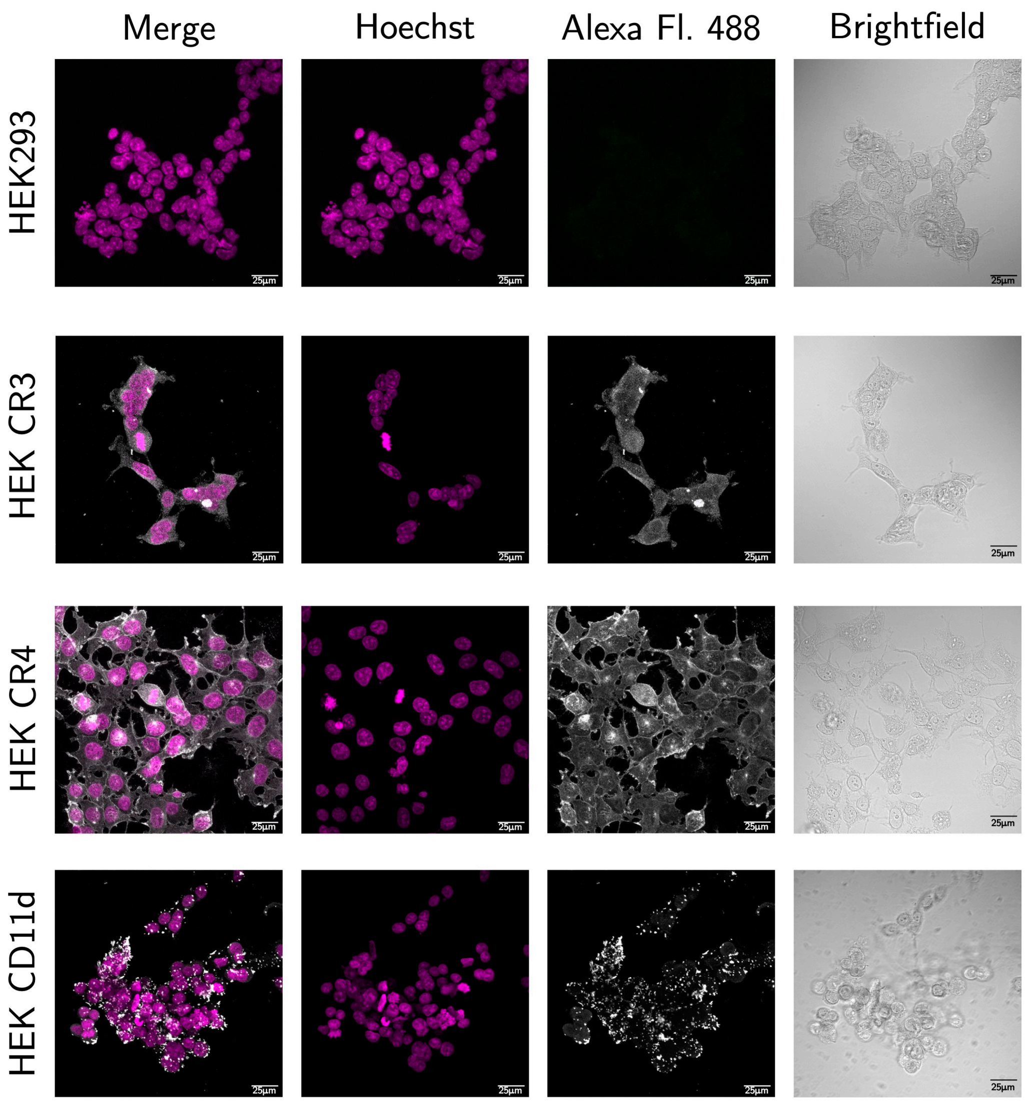

Figure S3. HEK cell lines imaged with confocal fluorescence microscopy at 60x magnification. In *magenta* cell nuclei and in *grey* receptors are shown. Cells were seeded to cell culture slides. After 24 h incubation, cells were washed with PBS and fixed with ice-cold methanol for 15 minutes. After washing three times with PBS, cells were blocked with 1 mg/mL BSA for 1 h at room temperature. Cells were stained in primary antibodies (see MATERIAL and METHODS section manuscript) diluted 1:1000 in 1 mg/mL BSA overnight at 4 °C. Cells were washed three times with PBS and incubated with secondary antibody (Alexa Fluor 488 anti-mouse and Alexa Fluor 488 anti-rabbit) 1:2000 in PBS for 1 h at room temperature. After washing three times with PBS, 1 µg/mL Hoechst solution was added and incubated for 5 minutes. Pictures were taken with an Olympus FV3000 microscope equipped with a x60 UPLANSApo objective. HEK293 do not express any β_2-integrin receptors, whereas HEK CR3 and HEK CR4 show a positive signal. In addition to the weak signal at the edges of the cell, the HEK CD11d cells also show aggregation of the antibody.

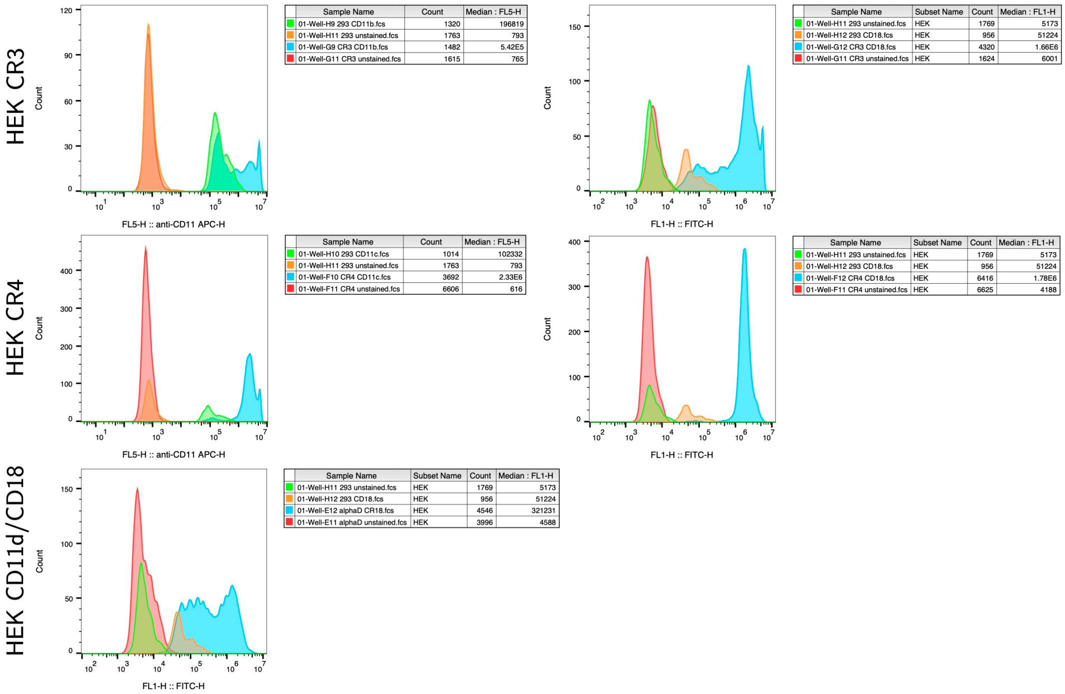

Figure S4. HEK cells were further characterized using flow cytometry. Cells were detached, transferred to a 96-well plate, and incubated for 30 minutes with fluorescently labelled antibodies before assessing with the Cytoflex flow cytometer (Beckman Coulter Life Science). The fluorescence was measured and in each sample 10’000 cells were counted. Data were analyzed using FlowJo software. HEK CR3, HEK CR4, and HEK CD11d all show a strong shift of the signal to the right in the staining for the β_2-subunit. HEK CR3 and HEK CR4 furthermore show a shift to the right in the staining for the α_M- and α_X-subunit, respectively.

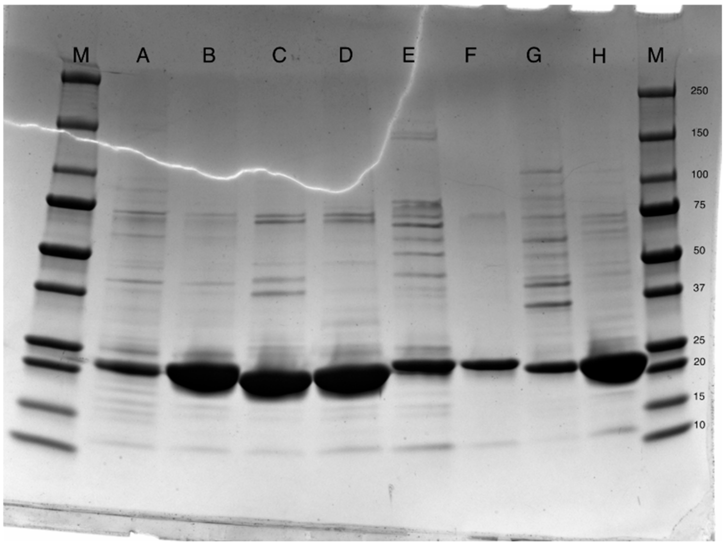

Figure S5. Uncropped, original gel of gel shown in Figure 1, main text.

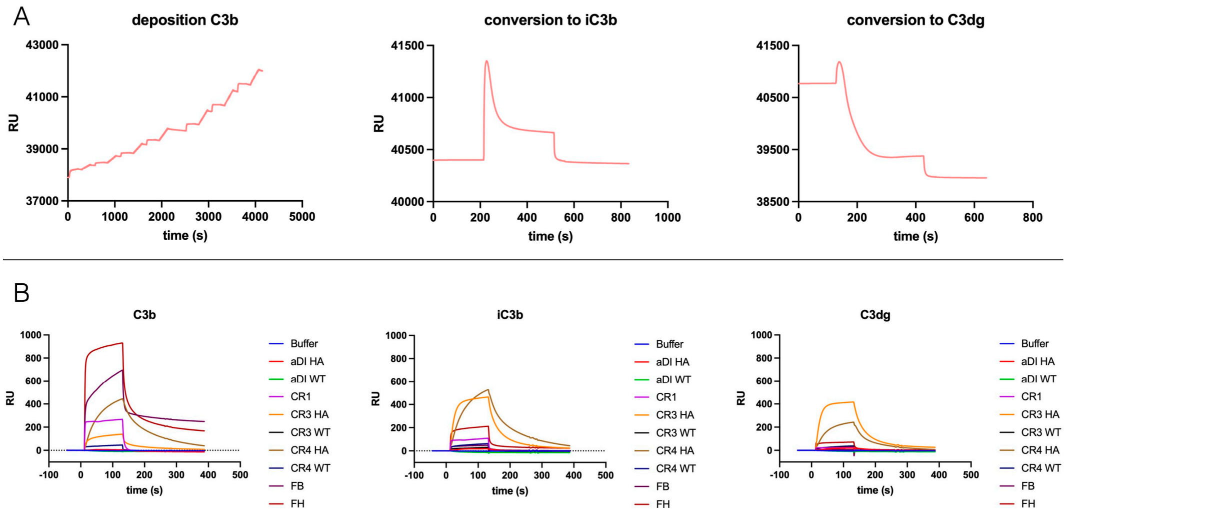

Figure S6. **A**: SPR Sensorgram of deposition of C3b and conversion to iC3b and C3dg. C3b was deposited on all three flow cells of the sensor chip via alternative convertase: FB and FD were injected alternately with C3. This was repeated several times until the desired surface density was reached. On one flow cell, C3b was cleaved using FI and FH to iC3b, and on one flow cell, C3b was cleaved using FI and CR1 to C3dg. **B**: As a control that C3b was cleaved successfully, single concentrations of different ligands were injected on all flow cells. To test if the cleavage of iC3b with FH and FI and C3dg with CR1 and FI was successful, single concentrations of different ligands were injected on all flow cells. FH and FB showed a huge binding to C3b as it was expected. Also, CR1 showed binding to C3b. The high-affinity variant of CR4 showed a huge binding to C3b, binding to iC3b and only low binding to C3dg, whereas the wild type did bind only very weak to C3b, weak to iC3b, and showed poor binding to C3dg. The CR3 high-affinity variant bound nicely to iC3b and C3dg, and to a small extent to C3b. On the other hand, CR3 wild type did not bind to C3b at all and bound slightly to iC3b and C3dg. CD11d α_DI high-affinity and wild type did not bind at all to C3b, iC3b, and C3dg.

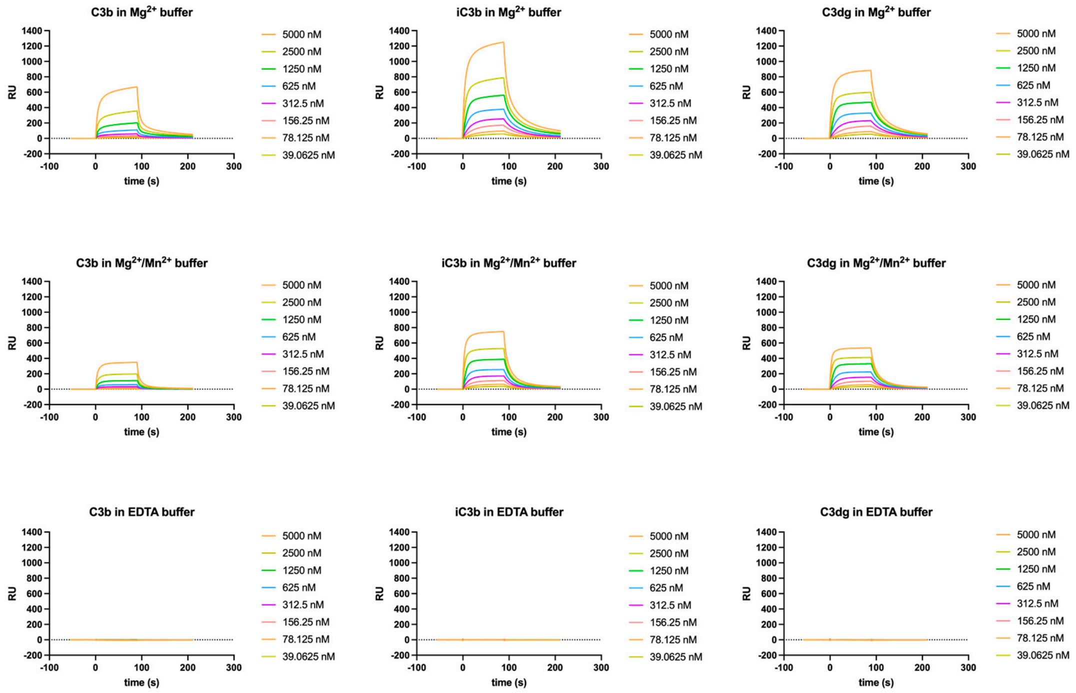

Figure S7. CR3 α_MI domain high-affinity binding to C3 fragments. SPR sensorgrams in HBST buffer supplemented with (from top to bottom) 1 mM $MgCl_2$, 1 mM $MgCl_2$ and 1 mM $MnCl_2$, and 5 mM EDTA. Mn^{2+} leads to lower RU values, but to increased on-rates. Binding in EDTA was inhibited, suggesting an involvement of the MIDAS in the binding interaction.

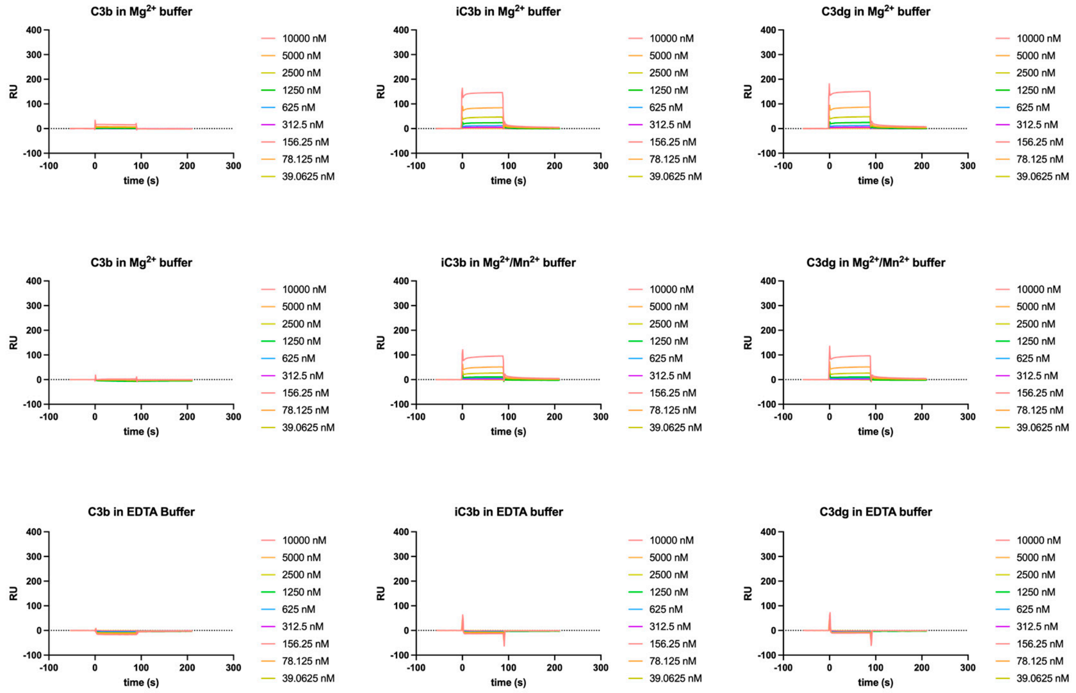

Figure S8. CR3 α_MI domain wild type binding to C3 fragments. SPR sensorgrams in HBST buffer supplemented with (from top to bottom) 1 mM $MgCl_2$, 1 mM $MgCl_2$ and 1 mM $MnCl_2$, and 5 mM EDTA. The CR3 α_MI domain binds to its main ligands iC3b and C3dg, which can be abolished using EDTA. No binding to C3b was observed.

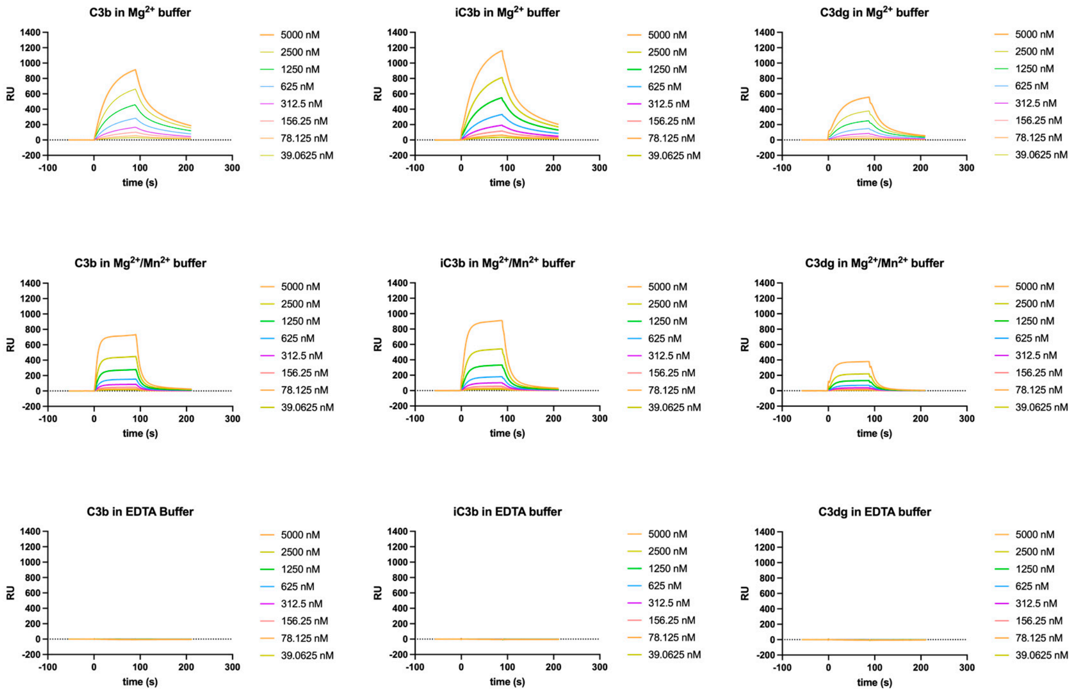

Figure S9. High-affinity CR4 α_XI domain binding to C3 fragments. SPR sensorgrams in HBST buffer supplemented with (from top to bottom) 1 mM $MgCl_2$, 1 mM $MgCl_2$ and 1 mM $MnCl_2$, and 5 mM EDTA. Mn^{2+} leads to lower RU values, but to increased on-rates. Binding in EDTA was abolished, indicating the involvement of the MIDAS.

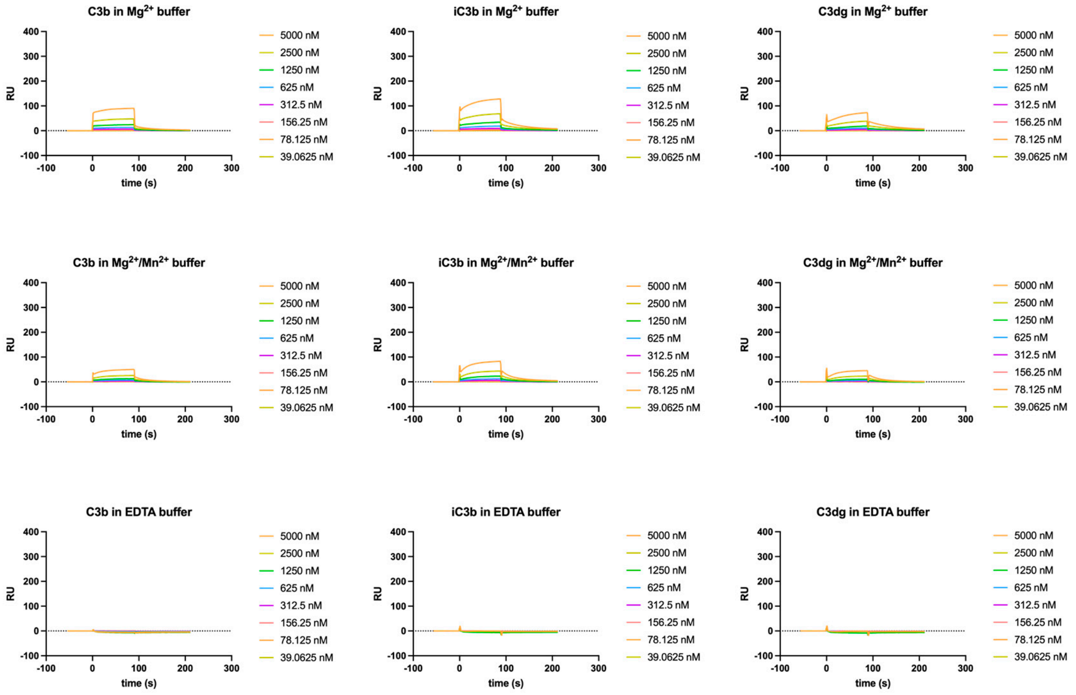

Figure S10. CR4 αXI domain wild type binding to C3 fragments SPR Sensorgram in HBST buffer supplemented with (from top to bottom) 1 mM $MgCl_2$, 1 mM $MgCl_2$ and 1 mM $MnCl_2$, and 5 mM EDTA.

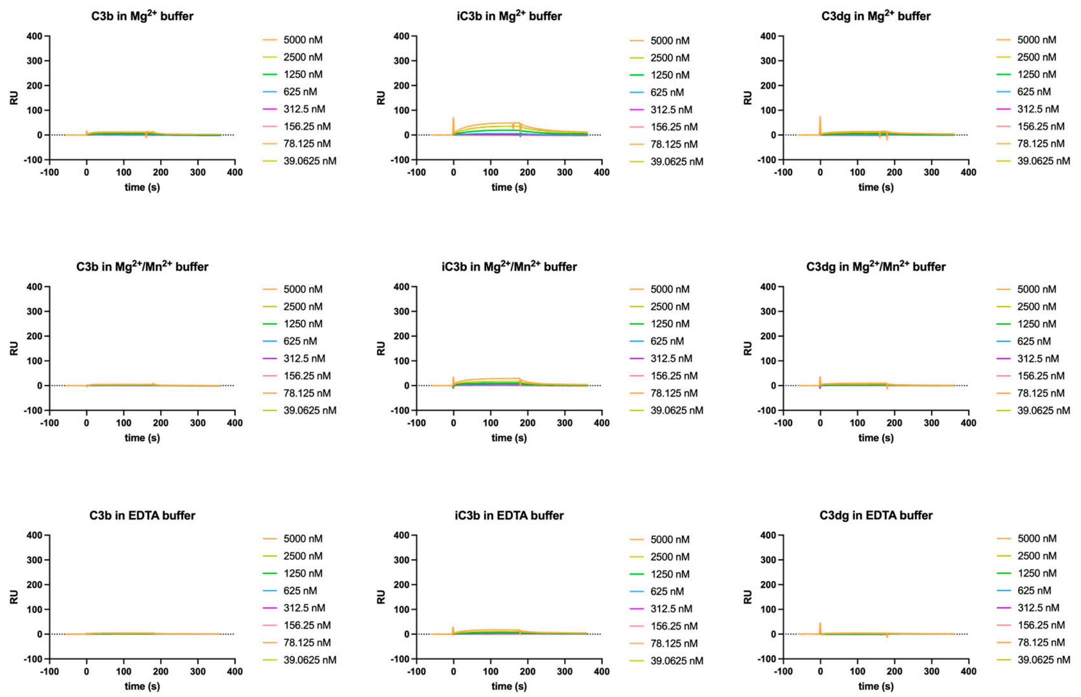

Figure S11. LFA-1 αLI high-affinity domain binding to C3 fragments. SPR sensorgrams in HBST buffer supplemented with (from top to bottom) 1 mM $MgCl_2$, 1 mM $MgCl_2$ and 1 mM $MnCl_2$, and 5 mM EDTA. No binding is observed.

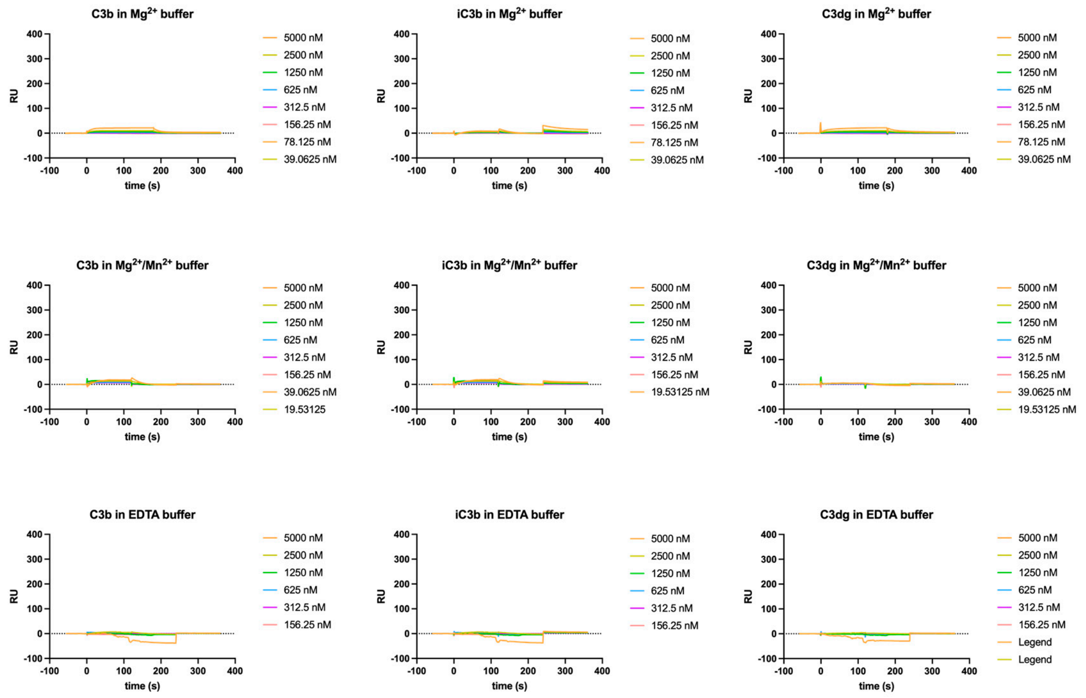

Figure S12. LFA-1 αLI wild type domain binding to C3 fragments. SPR sensorgrams in HBST buffer supplemented with (from top to bottom) 1 mM $MgCl_2$, 1 mM $MgCl_2$ and 1 mM $MnCl_2$, and 5 mM EDTA. No binding is observed.

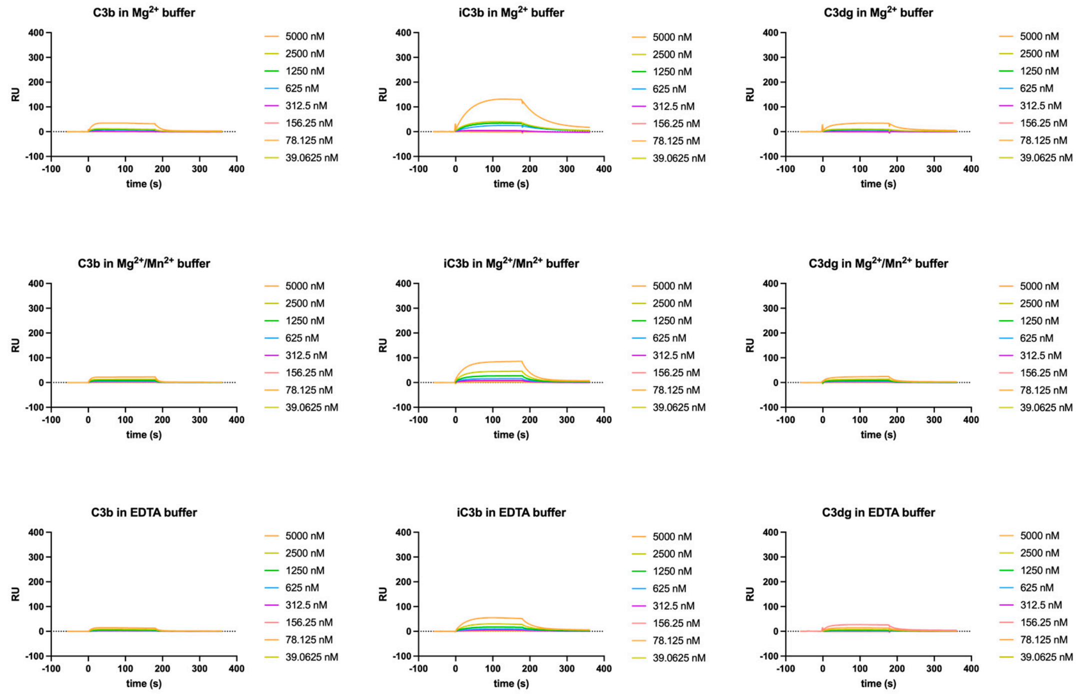

Figure S13. CD11d/CD18 α_DI high-affinity domain binding to C3 fragments. SPR sensorgrams in HBST buffer supplemented with (from top to bottom) 1 mM $MgCl_2$, 1 mM $MgCl_2$ and 1 mM $MnCl_2$, and 5 mM EDTA. CD11d/CD18 α_DI does not bind to C3b and C3dg. Binding to iC3b is not dependent on MIDAS or divalent cations.

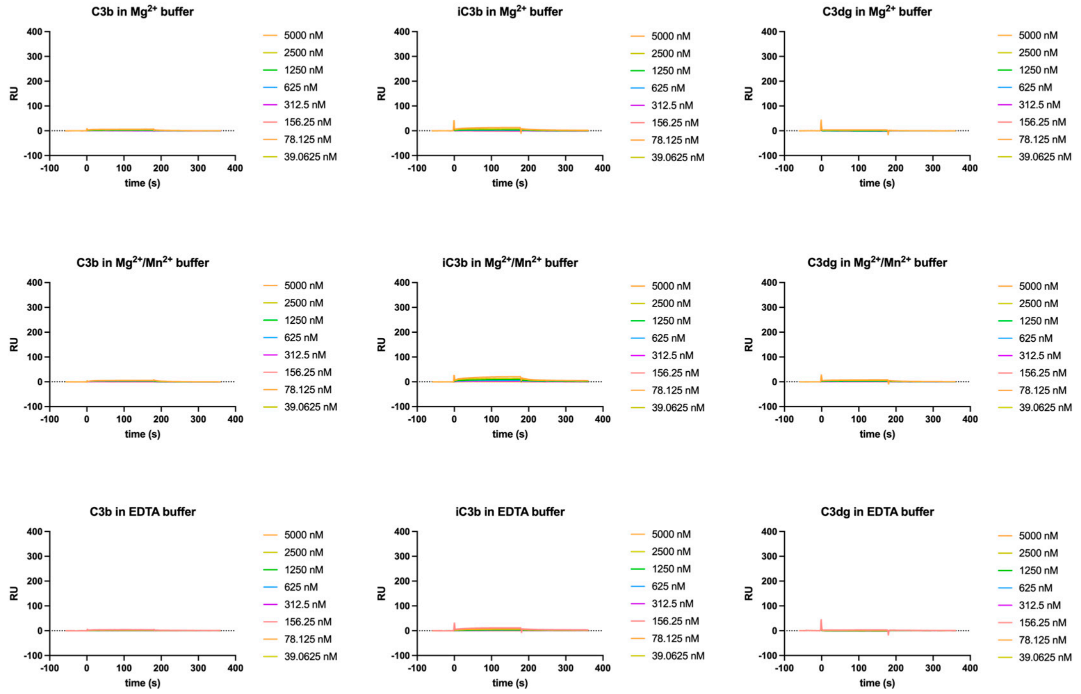

Figure S14. CD11d/CD18 α_DI wild type domain binding to C3 fragments. SPR sensorgrams in HBST buffer supplemented with (from top to bottom) 1 mM $MgCl_2$, 1 mM $MgCl_2$ and 1 mM $MnCl_2$, and 5 mM EDTA. No binding could be observed.

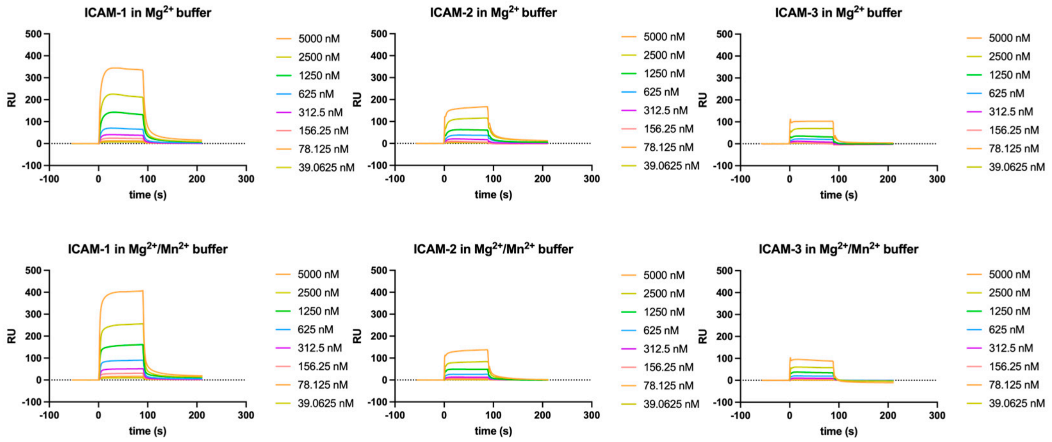

Figure S15. CR3 α_MI domain high-affinity binding to ICAM's. SPR sensorgrams in HBST buffer supplemented with (from top to bottom) 1 mM $MgCl_2$, and 1 mM $MgCl_2$ and 1 mM $MnCl_2$. CR3 α_MI binds to ICAM-1, -2, and -3.

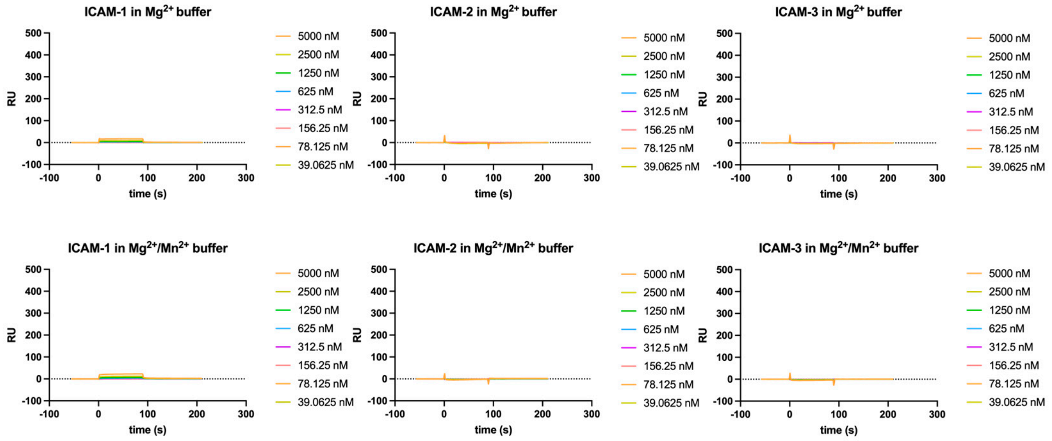

Figure S16. CR3 α_MI domain wild type binding to ICAM's. SPR sensorgrams in HBST buffer supplemented with (from top to bottom) 1 mM $MgCl_2$, and 1 mM $MgCl_2$ and 1 mM $MnCl_2$. No binding was observed.

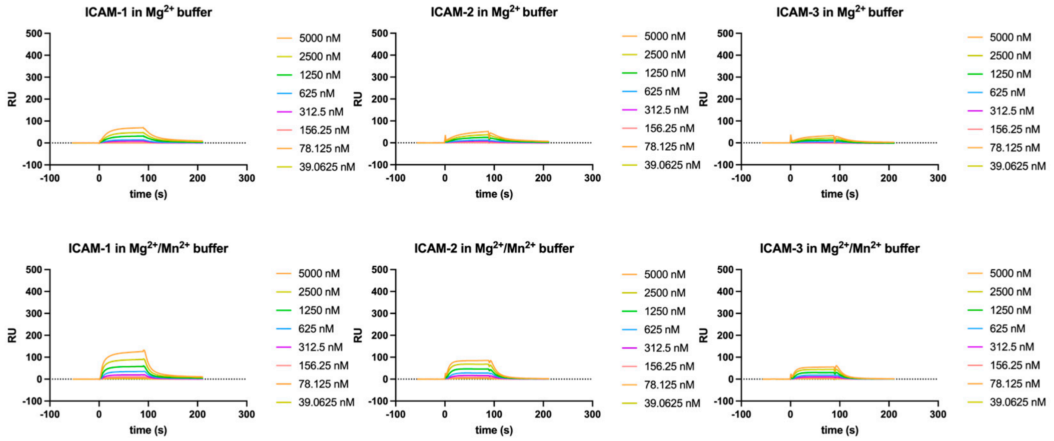

Figure S17. CR4 α_XI high-affinity domain binding to ICAM's. SPR sensorgrams in HBST buffer supplemented with (from top to bottom) 1 mM $MgCl_2$, and 1 mM $MgCl_2$ and 1 mM $MnCl_2$. Only weak binding to ICAM-1, -2, and -3 is observed.

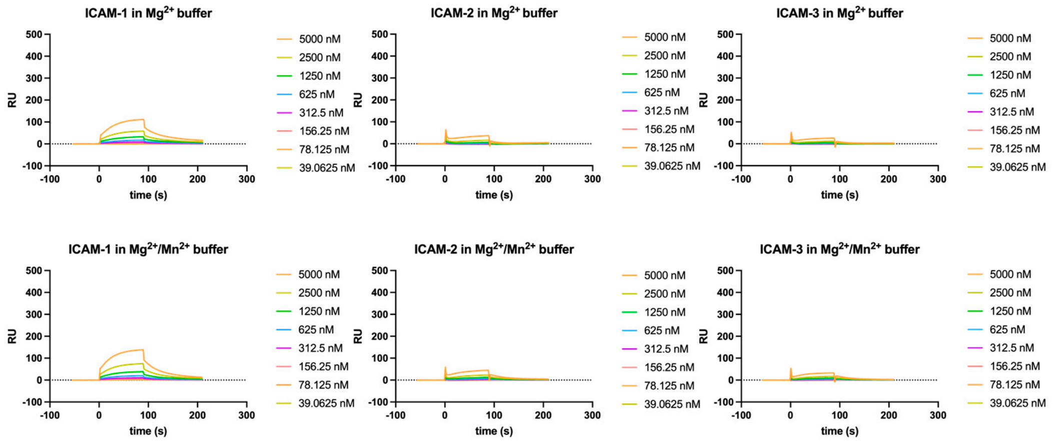

Figure S18. CR4 α_XI wild type domain binding to ICAM's. SPR sensorgrams in HBST buffer supplemented with (from top to bottom) 1 mM $MgCl_2$, and 1 mM $MgCl_2$ and 1 mM $MnCl_2$. Only CR4 α_XI binding to ICAM-1 is observed.

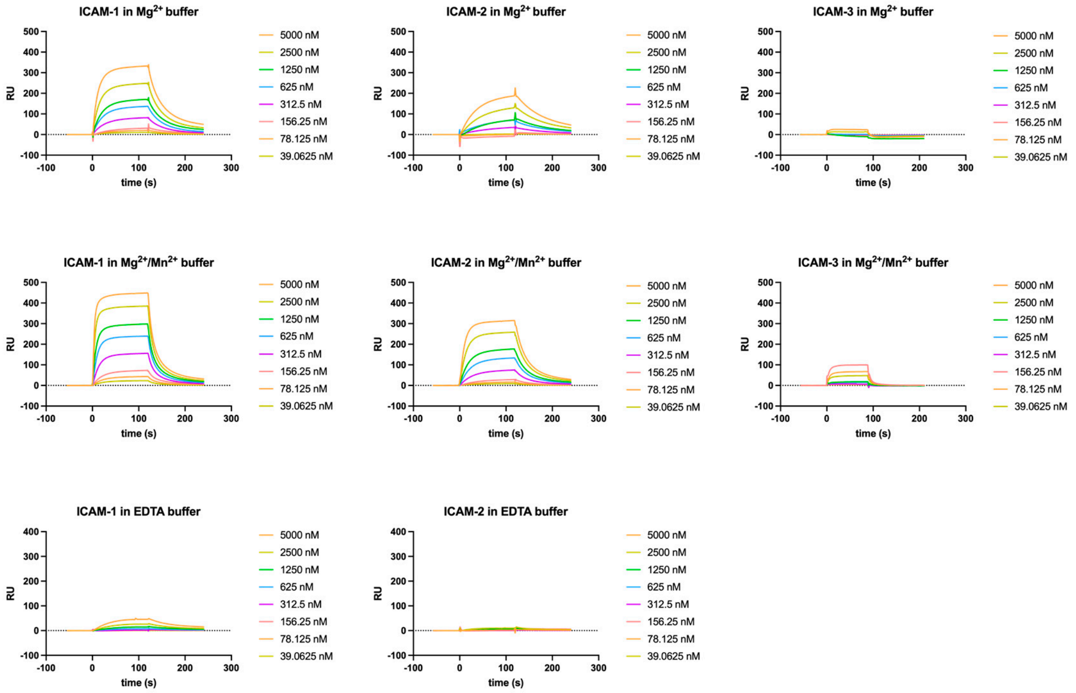

Figure S19. LFA-1 α_LI high-affinity domain binding to ICAM's. SPR sensorgrams in HBST buffer supplemented with (from top to bottom) 1 mM $MgCl_2$, 1 mM $MgCl_2$ and 1 mM $MnCl_2$, and 5 mM EDTA. LFA-1 α_LI HA binds to its ligands ICAM-1 and ICAM-2, which is enhanced in the presence of Mn^{2+} and diminished in the presence of EDTA. The binding to its ligand ICAM-3 can only be seen in the presence of Mn^{2+}.

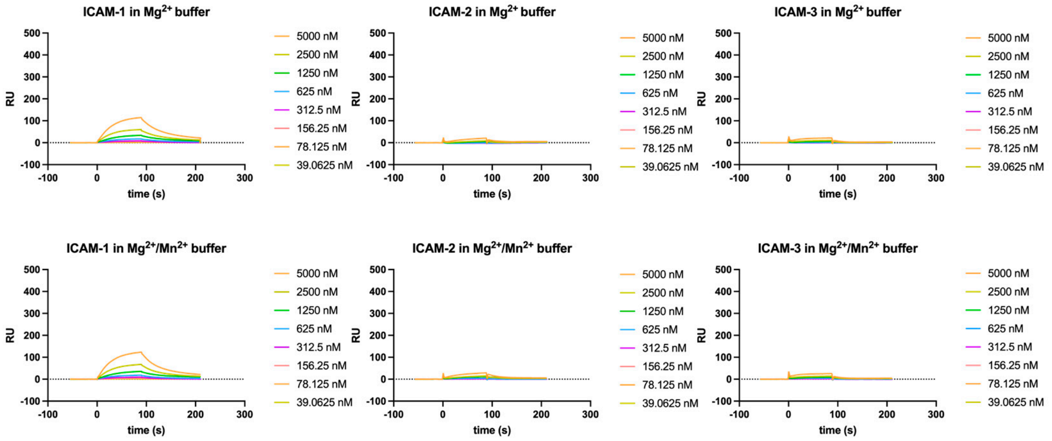

Figure S20. LFA-1 α_LI wild type domain binding to ICAM's. SPR sensorgrams in HBST buffer supplemented with (from top to bottom) 1 mM $MgCl_2$, and 1 mM $MgCl_2$ and 1 mM $MnCl_2$. Only binding to ICAM-1 can be observed.

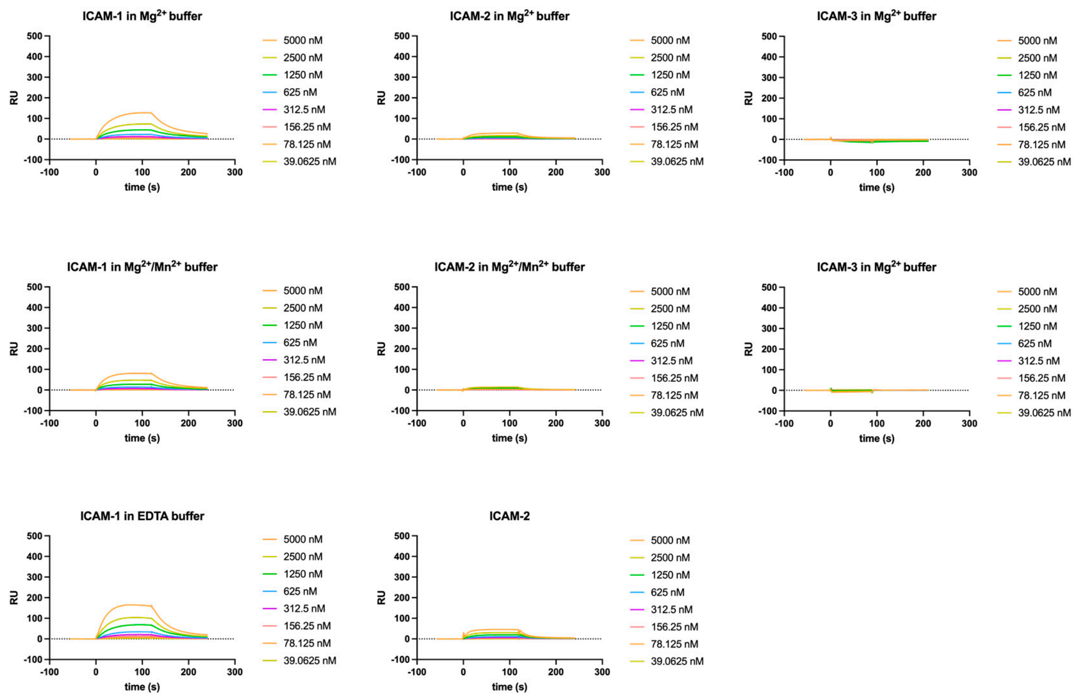

Figure S21. CD11d/CD18 α_DI high-affinity domain binding to ICAM's. SPR sensorgrams in HBST buffer supplemented with (from top to bottom) 1 mM $MgCl_2$, 1 mM $MgCl_2$ and 1 mM $MnCl_2$, and 5 mM EDTA. CD11d/CD18 α_DI binds to ICAM-1, but not to ICAM-2 and ICAM-3. Interestingly, this binding is not MIDAS-dependent.

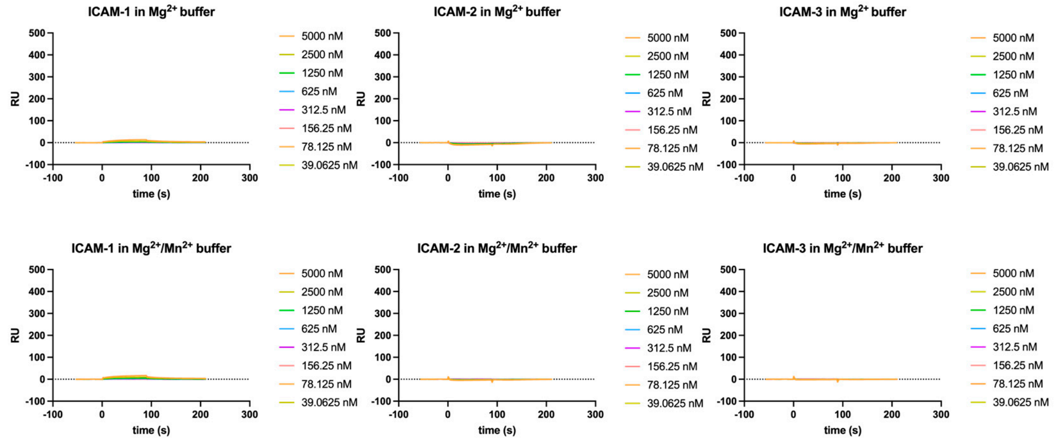

Figure S22. CD11d/CD18 α_DI wild type domain binding to ICAM's. SPR sensorgrams in HBST buffer supplemented with (from top to bottom) 1 mM $MgCl_2$, and 1 mM $MgCl_2$ and 1 mM $MnCl_2$. No binding is observed.

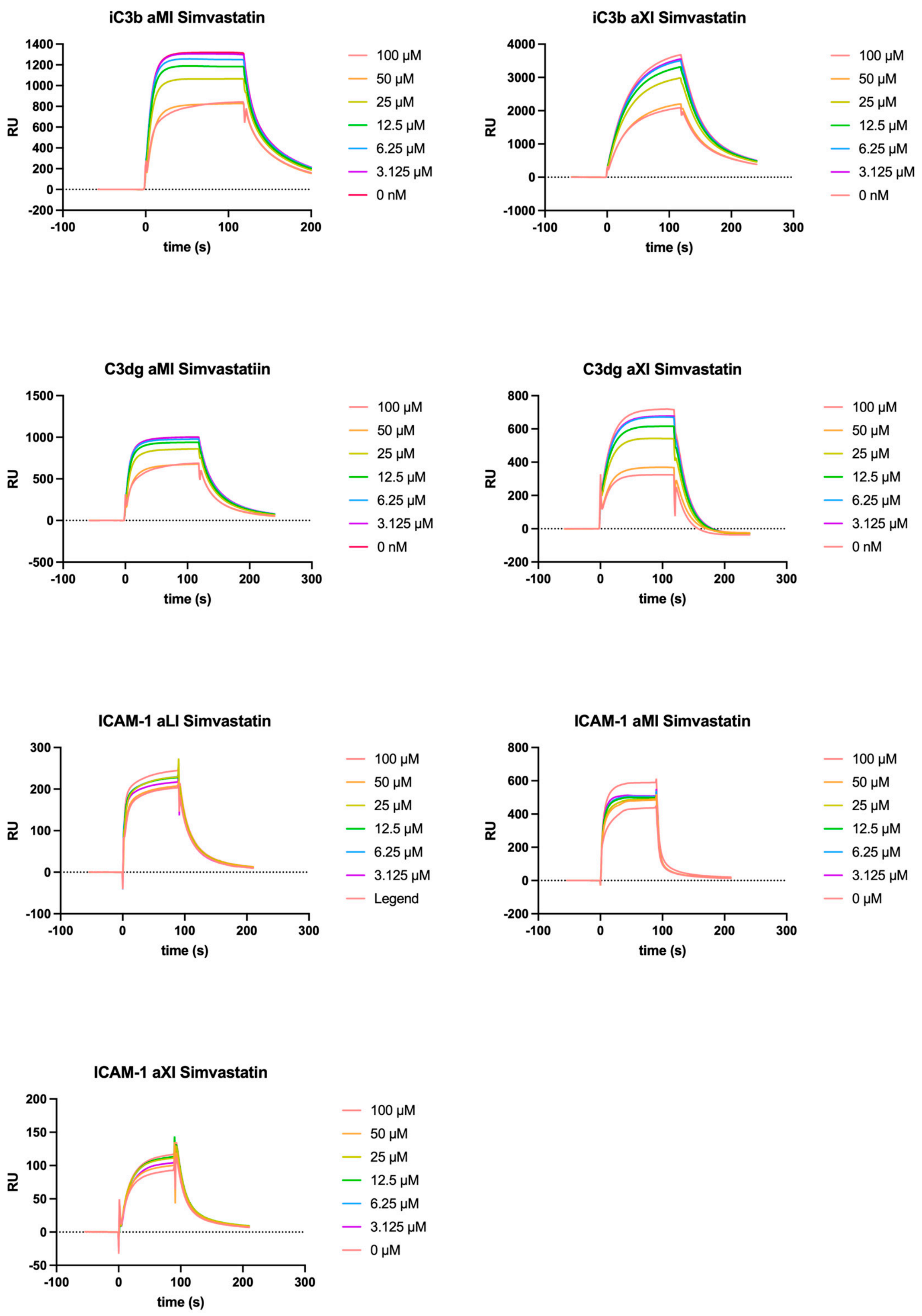

Figure S23. Competitive SPR using Simvastatin as an antagonist of β_2-integrins. The αI-domains in the high-affinity variant were preincubated at a fixed concentration with a dilution series of simvastatin ranging from 100 μM to 3.125 μM, running buffer was HBST supplemented with 1 mM $MgCl_2$ and 5 % DMSO. Simvastatin was able to inhibit binding of CR3 α_MI and CR4 α_XI to iC3b, C3dg, and ICAM-1 in a dose-dependent manner. LFA-1 α_LI binding to immobilized ICAM-1 was only slightly inhibited.

REFERENCES

1) Yakubenko VP, Yadav SP, Ugarova TP. Integrin αDβ2, an adhesion receptor up-regulated on macrophage foam cells, exhibits multiligand-binding properties. Blood 107 (2006) 1643–50. doi:10. 1182/blood-2005-06-2509.

2) Shimaoka M, Lu C, Palframan RT, von Andrian UH, McCormack A, Takagi J, et al. Reversibly locking a protein fold in an active conformation with a disulfide bond: integrin alphaL I domains with high affinity and antagonist activity in vivo. Proc Natl Acad Sci U S A 98 (2001) 6009–14. doi:10.1073/pnas.101130498.

3) Xiong JP, Li R, Essafi M, Stehle T, Arnaout MA. An isoleucine-based allosteric switch controls affinity and shape shifting in integrin CD11b α-domain. Journal of Biological Chemistry 275 (2000) 38762–38767. doi:DOI10.1074/jbc.C000563200.

4) Vorup-Jensen T, Ostermeier C, Shimaoka M, Hommel U, Springer TA. Structure and allosteric regulation of the alphaXbeta2 integrin I domain. Proc Natl Acad Sci U S A 100 (2003) 1873–8. doi:10.1073/pnas. 0237387100.
